# Long-range transport of airborne microbes over the global tropical and subtropical ocean

**DOI:** 10.1038/s41467-017-00110-9

**Published:** 2017-08-04

**Authors:** Eva Mayol, Jesús M. Arrieta, Maria A. Jiménez, Adrián Martínez-Asensio, Neus Garcias-Bonet, Jordi Dachs, Belén González-Gaya, Sarah-J. Royer, Verónica M. Benítez-Barrios, Eugenio Fraile-Nuez, Carlos M. Duarte

**Affiliations:** 1 Department of Global Change Research, Mediterranean Institute for Advanced Studies (IMEDEA), Spanish Council for Scientific Research – University of the Balearic Islands (CSIC-UIB), Esporles, Mallorca Spain; 2 Institute of Littoral, Environment and Societies (LIENSs), National Centre for Scientific Research (CNRS) – University of La Rochelle, La Rochelle, France; 3 King Abdullah University of Science and Technology, Red Sea Research Center, Thuwal, 23955-6900 Saudi Arabia; 4 Spanish Institute of Oceanography (IEO), Oceanographic Center of The Canary Islands, Santa Cruz de Tenerife, 38180 Spain; 5 Department of Physics, University of the Balearic Islands (UIB), Palma de Mallorca, Spain; 6 Department of Environmental Chemistry, Institute of Environmental Assessment and Water Research – Spanish Council for Scientific Research (IDAEA-CSIC), Barcelona, Catalonia Spain; 7 Department of Instrumental Analysis and Environmental Chemistry, Institute of Organic Chemistry - Spanish Council for Scientific Research (IQOG-CSIC), Madrid, Spain; 8 Institute of Marine Sciences - Spanish Council for Scientific Research (ICM-CSIC), Barcelona, Catalonia Spain; 9 Daniel K. Inouye Center for Microbial Oceanography, Research and Education, University of Hawaii at Manoa, Honolulu, USA; 10 OCEOMIC, Marine Bio and Technology S.L., Fuerteventura Technology Park, Puerto del Rosario, E35600 Spain

## Abstract

The atmosphere plays a fundamental role in the transport of microbes across the planet but it is often neglected as a microbial habitat. Although the ocean represents two thirds of the Earth’s surface, there is little information on the atmospheric microbial load over the open ocean. Here we provide a global estimate of microbial loads and air-sea exchanges over the tropical and subtropical oceans based on the data collected along the Malaspina 2010 Circumnavigation Expedition. Total loads of airborne prokaryotes and eukaryotes were estimated at 2.2 × 10^21^ and 2.1 × 10^21^ cells, respectively. Overall 33–68% of these microorganisms could be traced to a marine origin, being transported thousands of kilometres before re-entering the ocean. Moreover, our results show a substantial load of terrestrial microbes transported over the oceans, with abundances declining exponentially with distance from land and indicate that islands may act as stepping stones facilitating the transoceanic transport of terrestrial microbes.

## Abstract (web summary)

The extent to which the ocean acts as a sink and source of airborne particles to the atmosphere is unresolved. Here, the authors report high microbial loads over the tropical Atlantic, Pacific and Indian oceans and propose islands as stepping stones for the transoceanic transport of terrestrial microbes..

## Introduction

The atmosphere contains a substantial load of biological particles including bacteria and fungal spores^1^, which may be transported and deposited up to thousands of kilometres away from their source^2^. Average microbial abundances in the atmospheric boundary layer (ABL) around 1.9 × 10^4^ bacteria m^−3^ and 2.4 × 10^4^ fungal spores m^−3^ have been reported over land locations^3^. However, there are very few estimates of the abundance of airborne microbes at open oceanic locations^1, 2^. As a result of this lack of data, the role of the oceans as a sink and source of airborne microbes to the atmosphere is as yet unresolved^4^. The concentrations of total atmospheric particles and cloud condensation nuclei (CCN) over the ocean are substantially larger (few hundred per cm^3^)^5^ than those concentrations expected for microorganisms^1, 2^. Yet, bacteria and fungal spores may be more important for atmospheric chemistry and physics than what could be expected from their relatively low abundance. It has been hypothesised that the relative small quantity of microorganisms in the atmosphere could be specially important for the formation of ice nuclei (IN) at low latitudes where tropospheric temperatures are relatively high and abiotic particles are not efficient as ice nucleators^3^. In fact, most of the non-biological particles can be active as IN only when temperatures are below −10 °C or even in some cases they are active only at temperatures colder than −20 °C^6^. Among the biological particles able to contribute to ice-nucleation are mainly small bacteria and archaea, as well as organic compounds excreted by marine microorganisms, which were described as important IN over remote ocean locations^7^. While biological particles larger than 2 µm, such as fungal spores, are expected to act as giant CCN generating large drops with fast fall velocity^8^. Estimates of global bacterial emissions to the atmosphere at 40–1800 Gg dry weight y^−1^ assume a negligible oceanic contribution^4^. Yet, the oceans cover more than 70% of the Earth’s surface and therefore are likely the source and the final destination of a significant fraction of the biological particles suspended in the global atmosphere. In addition, evaluating the role of the oceans as a source and sink of airborne microbes as well as the potential for atmospheric transport of these microbes can provide important insights on the maintenance of microbial diversity^9, 10^, connectivity among terrestrial microbial communities and transoceanic spreading of microbes including human^11^ and crop^12^ pathogens.

Here we present a global survey reporting the abundance, fluxes and origin of the airborne microbes hovering over the Atlantic, Indian and Pacific Oceans at low latitudes carried out during the Malaspina 2010 Circumnavigation Expedition. Our data show that the oceans contribute a large fraction of the microbes found in the atmosphere. Moreover, we estimate that airborne microbes travel long distances over the oceans indicating that atmospheric transport of microbes may play a major role in the dispersal of surface marine microbes as well as in the intercontinental transport of their terrestrial counterparts.

## Results

### Abundance of airborne microbes over the World’s oceans

Abundances of airborne prokaryotes (bacteria and archaea) and unicellular eukaryotes (fungal spores, heterotrophic and autotrophic protists) were measured in 118 air samples collected along the Malaspina 2010 Circumnavigation Expedition that sailed the tropical and subtropical Atlantic, Indian and Pacific Oceans (between 40° S and 40° N, Supplementary Fig. 1).

**Figure 1:**
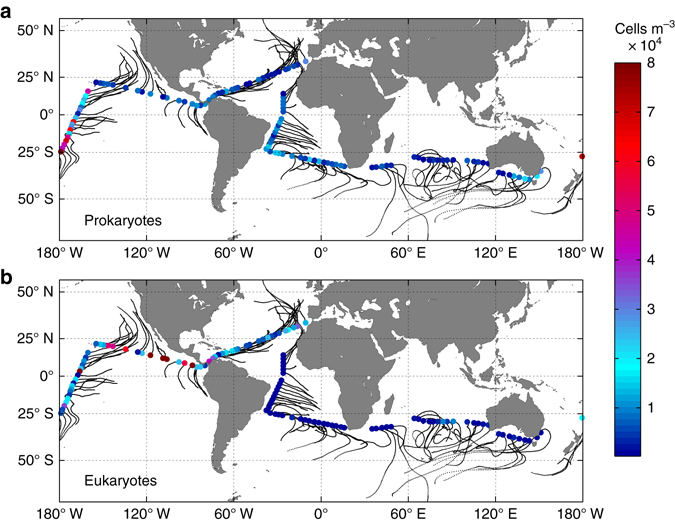
Abundances of airborne microbes over the ocean. Airborne prokaryotic **a** and eukaryotic abundances **b** over the Malaspina 2010 Circumnavigation Expedition. *Dots* correspond to sampled locations and *black lines* correspond to backward trajectories of air masses modelled for 5 days previous to the air sampling starting at 10 m above sea level

Microbial abundances in the ABL over the open ocean varied widely among the sampled locations, ranging between 5 × 10^2^ and 8 × 10^4^ cells m^−3^ of air for prokaryotes (median 6.7 × 10^3^ cells m^−3^, Fig. 1a), and from 1 × 10^2^ to 1.8 × 10^5^ cells m^−3^ for unicellular eukaryotes (median 3.2 × 10^3^ cells m^−3^, Fig. 1b). Median values were consistent with estimated microbial abundances over the ocean from global models^3, 4^, while direct microbial counts from previous studies over marine coastal locations were usually closer to the highest prokaryotic abundances^13, 14^ and to the median values of eukaryotes^15^ reported in this work. The number of airborne microorganisms is small (<0.1%) if compared with the number of aerosols in the oceanic atmosphere, which is dominated by submicron size aerosols^5^. However, the size-dependent distribution of surface and mass (volume) of the aerosol pool do not generally follow the distribution of number of aerosols, but shows a maximum for micron-sized aerosols^16, 17^. In the oceanic atmosphere, biological aerosols (prokaryotes and eukaryotes) may play a disproportionate role in those processes dependent on the surface and mass of the aerosol population, such as the formation of IN, the absorption/reflection of solar radiation, or especially in terms of atmospheric deposition fluxes of organic matter. Indeed, settling velocities for large aerosols (several microns) are known to be larger than those of submicron aerosols^18^, and the overall deposition flux of organic matter is strongly dependent on the fraction of large aerosols.

### Microbial contribution of land and oceans to the atmosphere

**Figure 2:**
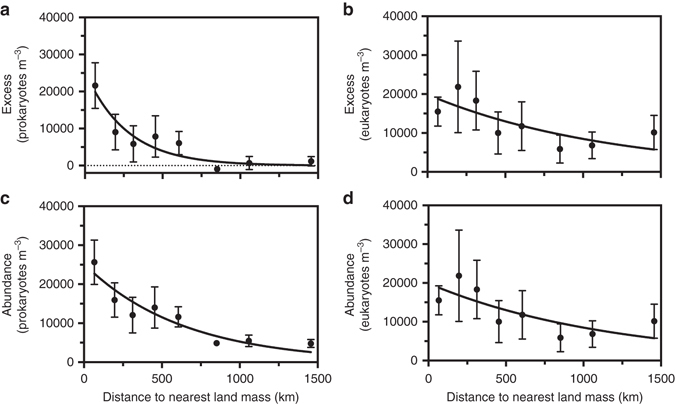
Relationship between the excess and the abundance of microbes with distance to land. The excess of **a** prokaryotes and **b** eukaryotes, and the airborne abundances of **c** prokaryotes and **d** eukaryotes versus the distance (*D*, km) to the nearest land mass. The distance is grouped into 8 bins. *Black dots* represent the mean values and error bars represent the standard error of the mean (SEM). The *solid lines* show the fitted exponential models suggested by the Akaike information criterion (*AIC*) and gives for: **a** prokaryotic excess (cells m^−3^) = 25454e^(−0.0037*D*)^; *R* ^2^ = 0.87; **b** eukaryotic excess (cells m^−3^) = 19881e^(−0.00085*D*)^; *R* ^2^ = 0.58; **c** prokaryotic abundance (cells m^−3^) = 25217e^(−0.0016*D*)^; *R* ^2^ = 0.88; **d** eukaryotic abundance (cells m^−3^) = 19883e^(−0.00085*D*)^; *R* ^2^ = 0.58

We constrained the potential role of the surface ocean as a source of atmospheric prokaryotes and unicellular eukaryotes using a simple model considering only spray inputs into the atmosphere and losses by deposition estimated using previously reported parameterisations^19, 20^. Thus, obviating advection, the concentration of airborne microbes that can be provided by the surface ocean would be that in the steady state between spray inputs and deposition. This depends mainly on the abundance of microbes in surface water and wind speed, and to a lesser extent on other parameters such as humidity and temperature in the used parameterisations. These estimated ranges are towards the low range of our estimates. In fact, 32% of the sampled locations greatly exceeded the prokaryotic abundances calculated from purely local oceanic contributions estimated from the in situ environmental conditions and abundances in surface waters, or to put in another way, 32% of the samples showed a high prokaryotic excess. In contrast, the eukaryotic cells largely exceeded the calculated marine contribution in all of the samples, suggesting that air samples included allochthonous eukaryotes advected from other locations. Many samples presenting prokaryotic abundances higher than the estimated oceanic range were located in the vicinity of the numerous islands found in the West and Central Pacific regions (Fig. 1, regions described in Supplementary Fig. 1). The abundance of airborne prokaryotes observed close to islands and continents exceeded the predicted oceanic contribution by, on average, 2.5 × 10^4^ cells m^−3^, well in the range of the average abundance of 2 × 10^4^ cells m^−3^ reported over land locations^3^. This prokaryotic excess was higher near the coast and decreased exponentially with distance away from the nearest land mass (Fig. 2), suggesting that the higher prokaryotic abundances as compared to those expected from a local marine contribution were mainly of terrestrial origin. Indeed, the absence of correlation between airborne microbial abundances and other atmospheric and oceanographic parameters, such as wind speed, waves and chlorophyll-*a* data, suggests that those factors play a minor role as compared to terrestrial inputs in controlling the variability observed in airborne microbial abundances among locations (Supplementary Table 1 and Supplementary Fig. 2).

### Atmospheric transport of microbes

Unicellular eukaryotic abundances also showed a decreasing trend when moving away from land (Fig. 2). The maximum eukaryotic values were found in the North Atlantic and East Pacific, where most of the prokaryotic abundances were substantially lower than those of eukaryotes even at locations far from the coast (Supplementary Fig. 3a). These results can be explained because the North Atlantic and East Pacific regions, sampled during summer and spring, respectively, were probably affected by the typical events of African dust exports to the Atlantic Ocean during summer and Asian dust exports into the Pacific during spring^21^. In fact, Moderate-Resolution Imaging Spectroradiometer (MODIS) reported high values of aerosol optical depth (AOD) over the East Pacific and Atlantic during May and especially highest over the Atlantic during June 2011^22^. Moreover, the expected terrestrial origin of eukaryotes is consistent with the lack of phytoplanktonic microorganisms observed under the microscope in the analysed air samples, with the high fungal spore abundances previously found in coastal areas collected during dust storms^23^, and with the fact that fungal spores made the bulk of the eukaryotic counts. In addition, the observed increment in the ratio between eukaryotes and prokaryotes with distance to land (Supplementary Fig. 3b, c) is in agreement with the observed ability of some spores to remain suspended in the air for extended periods of time^24^.

Despite the strong loss of atmospheric microbes with the distance to the coast, the abundance of prokaryotes at locations 1000 km away from land was about 20% of that found at locations close to the coast (Fig. 2). This value is somewhat smaller than the estimated 30–50% of total aerosols able to exceed distances of 1000 km from tropical and temperate sources^25^. Moreover, at these locations the relationship between prokaryotic excess and distance to the nearest land mass predicted that 2.5% of prokaryotic excess remain suspended in the atmosphere, which means that a fraction of the allochthonous input of microbes has the potential for long-range or even transoceanic transport.

### Sources of airborne microbes

**Figure 3:**
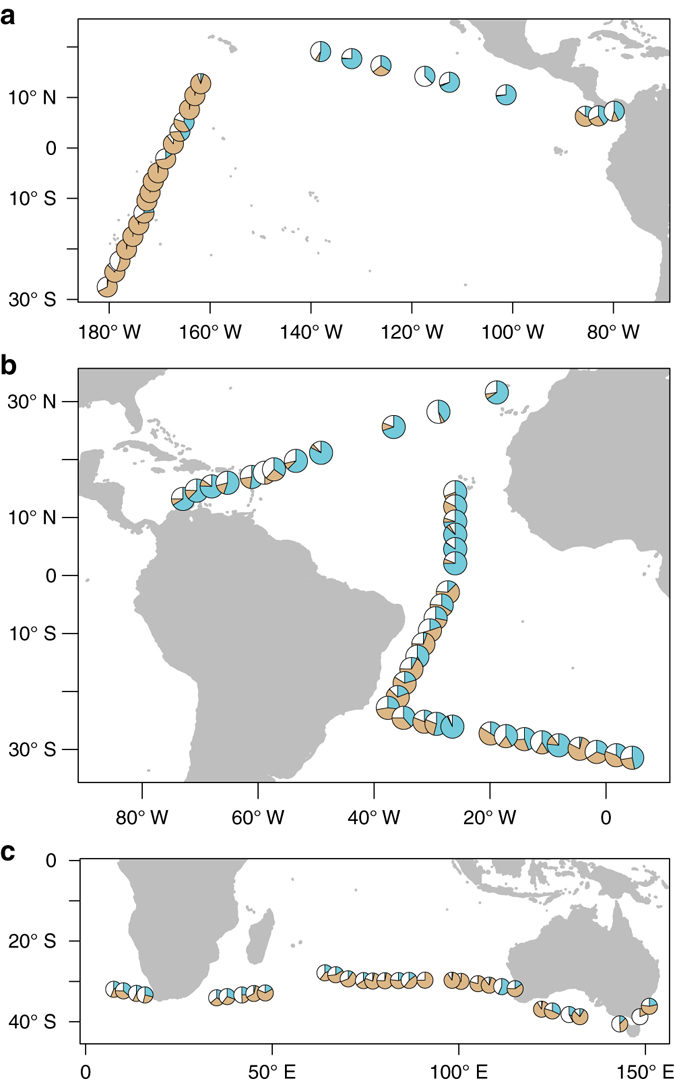
Origin of the prokaryotic sequences. The pie charts represent the estimated proportion contributed by marine (*blue*), terrestrial (*brown*) and unknown (*white*) sources for the 99 sampled locations over the Pacific **a**, Atlantic **b** and Indian **c** oceans where we could get sequence information. Please note that the different panels have different scales. Data available in Supplementary Table 2

Independent confirmation on the origin of airborne microbes (Fig. 3) was obtained by comparing the phylogenetic affiliations of airborne prokaryotes with those found in surface seawater samples collected in the Malaspina Expedition (Supplementary Fig. 1), and in different datasets from marine and terrestrial ecosystems (see Methods). Overall, airborne microbial assemblages could be explained by a mixture of about 25% of microbes from marine origin with about 42% of terrestrial microorganisms (median values). A relatively large fraction (median 24%) could not be explained by our reference data set suggesting either terrestrial sources or marine contributions from remote areas not represented in our reference data set. This indicates that long-range dispersal of airborne prokaryotes and substantial inputs from non-marine sources shape the composition of airborne microbial assemblages. The regions where the largest excess of prokaryotes relative to the potential marine contribution were estimated (West and Central Pacific, Supplementary Fig. 4a) showed the lowest percentage of marine sequences (1% and 2% respectively, Fig. 3b, Supplementary Fig. 4b), a clear dominance of terrestrial sequences (around 90%, Fig. 3b, Supplementary Fig. 4c), and a small percentage of sequences not found in our marine reference data set (5% and 8%, respectively, Fig. 3b, Supplementary Fig. 4d). This is consistent with a high excess of prokaryotes predicted over these regions, where most of the air samples were located relatively close to land resulting in the dominance of advective flows (Supplementary Fig. 4e). Unexpectedly, terrestrial prokaryotic sequences and those not present in the databases comprised more than half of the sequences detectable in the South Atlantic and Indian Oceans, two regions located far from land and apparently influenced by air masses of marine origin (Supplementary Fig. 1). In fact, marine sequences only represented 35% and 15% of the total of those detected over the South Atlantic region and the Indian Ocean, respectively (Fig. 3a, c, Supplementary Fig. 4b). The high contribution of terrestrial sequences relative to those of marine origin is consistent with the high concentrations of urban aerosols found over these regions during the Malaspina 2010 Circumnavigation Expedition^26^. This was probably due to plumes of particles released from land and vertically mixed at altitudes above the boundary layer, which have been described as a frequent phenomenon over the tropical Indian Ocean^27^. Indeed, most of the air trajectories in the Indian Ocean and some of those in the South Atlantic reached altitudes higher than 1000 m that typically exceed the altitude of the ABL (Supplementary Figs. 5 and 6). At these altitudes airborne particles can be advected from land to the ocean where they can easily mix down to lower altitudes because the thermal inversion at the top of the boundary layer is weaker in the Indian Ocean than in other locations^27^, enhancing the bacterial exchange at the top of the boundary layer. However, around 30% of the community composition found over the Indian Ocean could not be attributed to either terrestrial or marine sources, suggesting a significant contribution of remote sources not included in our reference dataset. The Central Atlantic was characterised by low prokaryotic abundances consistent with those expected from a marine origin and represented the highest percentages of marine sequences (Fig. 3a, Supplementary Fig. 4b). Back trajectory analysis showed that the air masses sampled in the Central Atlantic were originated from land locations. However, these stations were among those situated farthest away from the closest land point, which could explain the dominance of marine phylotypes in these samples. The North Atlantic region showed a prevalence of marine sequences (median slightly over 60%), in agreement with the observed abundances of airborne prokaryotes that were close to those expected under equilibrium with the surface ocean (on average −1160 ± 1760 prokaryotic cells below or above equilibrium) in 77% of the North Atlantic locations, while the East Pacific region showed a prevalence of marine and undetermined contributions (medians slightly over 35%, Fig. 3, Supplementary Fig. 4). This high percentage of undefined sequences could be associated with the high numbers of observed eukaryotes, probably coming from land, over this region, suggesting that at least a portion of these undefined sequences can correspond to prokaryotes derived from long-range transport from land locations. Moreover, a large percentage of fungal mitochondrial sequences was detected in many samples while plastids and other marine eukaryotic sequences that frequently appear in 16S rDNA surveys made a very low contribution to almost all of the samples (Supplementary Table 2). This is in agreement with the microscopic observations where we found abundant fungal spores but very few cells of phytoplankton or other marine eukaryotes.

The whole-community approach used to estimate the relative contribution of marine versus terrestrial OTUs did not use the phylogenetic assignment of each sequence, and therefore is free of dubious assumptions as to the attribution of a terrestrial or marine origin to each individual sequence. However, the comparison among airborne and ocean surface communities yields strong indications as to the terrestrial origin of a large fraction of the airborne sequences. This includes the prevalence of Betaproteobacteria, Actinobacteria and Bacilli found in airborne samples (Supplementary Fig. 7). These groups are practically absent in the reference surface seawater samples collected during the Malaspina Expedition (Supplementary Fig. 7) and are generally rare in marine environments elsewhere, but abundant in freshwater and terrestrial environments^28^.

### Exchange of microbes between the ocean and the atmosphere

The role of the oceans as a sink and source of airborne microbes to the atmosphere was evaluated by estimating deposition and emission fluxes in the sampled locations. Dry deposition fluxes ranged between 1 × 10^4^ and 6 × 10^6^ prokaryotic cells m^−2^ day^−1^ and between 1 × 10^4^ and 2 × 10^7^ eukaryotes m^−2^ day^−1^ (Supplementary Table 3), and were generally higher (Wilcoxon matched-pairs signed rank test, *P*_prokaryotes_ = 0.052, *P*_eukaryotes_ < 0.0001) than the corresponding emission fluxes, which ranged between 1 × 10^3^ and 2 × 10^6^ prokaryotic cells m^−2^ day^−1^ and between 1 and 2000 eukaryotes m^−2^ day^−1^ (Supplementary Table 3). The prevalence of net depositional microbial fluxes indicates that the ocean mainly acts as a sink for airborne microbes, largely originating from terrestrial sources. There were 12 rain precipitation events recorded during the track of the Malaspina Expedition, but only 8 of these events were relatively close in time to 10 of the air sampled locations (Supplementary Table 1) although rain events never coincided with sampling operations. We estimated wet deposition of prokaryotes and eukaryotes for the sampling sites closest to the rainfall events (see Supplementary Methods). Although these estimates were substantially higher than dry deposition fluxes, consistent with the high efficiency of rain to scavenge aerosols^29^, only one location associated to a rain event showed a large decrease in both airborne prokaryotic and eukaryotic abundances as compared to the previous sample, while the remaining nine locations close to rain events showed minor decreases or even increasing abundances for both prokaryotes and eukaryotes as compared to the values observed in the previous sample (Supplementary Fig. 8). Episodic events of wet deposition may have a large impact on the local abundance of airborne microbes over short periods of time in the range of hours. However, rain events were rare during the Malaspina Expedition and therefore wet deposition is unlikely to have a measurable effect in our measurements.

Dry deposition fluxes indicate that 50% of prokaryotic and eukaryotic cells settled after a median of 17 and 3.5 days, respectively. Using a dispersion model^30^, we estimated that 50% of the airborne prokaryotes and eukaryotes remain suspended after being transported over 22000 and 6000 km into the ABL, respectively (Supplementary Table 3). This is sufficient to allow intercontinental transport depending on wind direction. It must be noted that over some regions of the ocean where rain events are more frequent the residence times are expected to be shorter than those reported in this study^31^ due to the enhanced removal by wet deposition resulting in reduced residence times and potential transport distances.

### Global marine contribution of microbes to the atmosphere

Our results indicate that the atmosphere over the open ocean harbours abundances of airborne prokaryotes between 5 × 10^2^ and 8 × 10^4^ cells m^−3^. By constraining prokaryotic abundances in the surface ocean to a typical range from 0.5 × 10^6^ to 1.5 × 10^6^ cells ml^−1^ and using wind speeds larger than 6 m s^−1^, representative of open-ocean conditions^32^, we can attempt to estimate the magnitude of oceanic contributions to global airborne microbial abundance. Using these constrains and the same steady-state model used to calculate the oceanic contribution to microbial abundance in our samples, we estimate that prokaryotic emissions from the ocean support abundances of about 3 × 10^3^ to 1.3 × 10^4^ cells m^−3^ (Supplementary Fig. 9) while higher abundances are typically found close to islands and land masses showing substantial inputs of terrestrial microbes. However, the ocean covers more than 70% of the Earth’s surface and the calculated atmospheric boundary layer over the oceans is on average thicker than over land^33^, which results in oceanic prokaryotes contributing roughly between 33 and 68% of the prokaryotes present in the global atmosphere. Our data also show that terrestrial microbes reach far out into the ocean and can represent a large fraction of the microbes found over oceanic locations at least at the low to mid-latitudes surveyed here.

**Figure 4:**
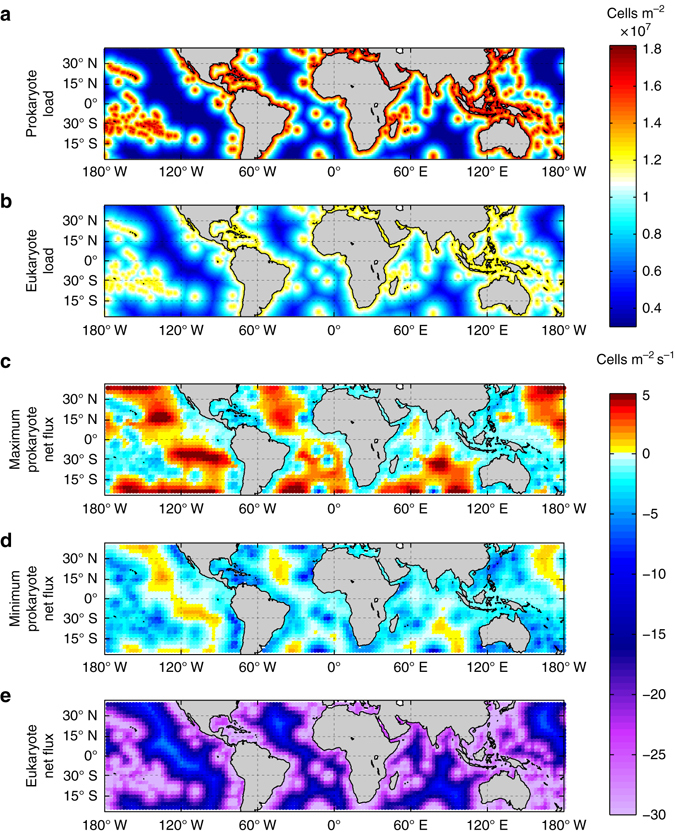
Microbial loads and air-sea exchange fluxes over the global tropical and subtropical ocean. Prokaryotic load **a** and eukaryotic load **b** over the ocean extension compressed between 40° S and 40° N with resolution of 1° × 1°; maximum net fluxes of prokaryotes considering high spray fluxes **c** and low spray fluxes **d**; and net fluxes of eukaryotes **e** considering high spray fluxes (the differences between net fluxes of eukaryotes using high or low spray fluxes were negligible) with resolution of 2.5° × 2.5°. Negative values indicate net deposition fluxes

Upscaling over the ocean the load of airborne microbes across a grid of 1° × 1° between 40° S and 40° N using the exponential model fit with distance from the nearest land mass (Supplementary Fig. 10) we estimated a total load of 2.2 × 10^21^ prokaryotes and 2.1 × 10^21^ eukaryotes over the global ocean (Fig. 4). Yet, these stocks are the result of a constant exchange involving a much larger number of microbes. We estimated that gross emission fluxes of airborne microbes over the ocean between 40°S and 40°N ranged from 7.5 × 10^21^ to 2.3 × 10^22^ prokaryotes y^−1^ and from 7.5 × 10^18^ to 2.3 × 10^19^ eukaryotes y^−1^, while gross estimated deposition fluxes reached 2.3 × 10^22^ prokaryotes and 1.6 × 10^23^ eukaryotes y^−1^, resulting in a net depositional fluxes of up to 1.5 × 10^22^ prokaryotes and about 1.6 × 10^23^ eukaryotes y^−1^ (Fig. 4). The net deposition fluxes into the ocean were equivalent to a carbon mass of up to 2.0 × 10^−4^ Tg C y^−1^ for prokaryotes and about 2.1 Tg C y^−1^ for eukaryotes, comparable to the emissions calculated from land sources^4^.

In summary, the results presented improve on prior attempts at producing global budgets of microbial abundance in the atmosphere by providing reliable measurements of airborne microbial abundance at offshore locations, constraining the marine versus terrestrial origin of airborne microbes over the ocean, and demonstrating the role of islands in maintaining elevated levels of airborne organisms over the ocean accounting for the high concentrations observed in the subtropical Pacific (Fig. 1). Moreover, our calculations show that the residence times of 17 and 3.5 days may suffice to transport airborne prokaryotes and eukaryotes over thousands of kilometres. Hence, the global atmosphere plays an important role both in the intercontinental transport of terrestrial microorganisms and in the dispersal of oceanic microbes across distant oceanic regions, with islands acting as stepping-stones for cross-oceanic transport of terrestrial microbes.

## Methods

### Study area

Samples of airborne microbes were collected along the Malaspina 2010 Circumnavigation Expedition^34^ on board the R/V *Hespérides* from December 2010 to July 2011 collecting samples over 118 locations across the Atlantic, Pacific and Indian Oceans (Supplementary Fig. 1). According to the atmospheric circulation of the study area, characterised using atmospheric monthly field data obtained from the NCEP/NCAR atmospheric reanalysis^35^, most samples were taken near the Inter Tropical Convergence Zone (ITCZ) where the winds are relatively weak and typically prevailing from the east. The trade winds converging along the ITCZ come from NE in the Northern Hemisphere and from SE in the Southern Hemisphere. The position of the ITCZ (around the equator) migrates from the Southern to the Northern Hemisphere between January and July over the Indian and Pacific Oceans but it does not significantly change in the Atlantic Ocean during the same period of time. The North Atlantic region is characterised by the presence of strong northeasterly winds, enhancing the transport of biological particles across the Atlantic from Europe. When the South Atlantic region was sampled (February 2011) the wind direction was mainly from SE, and from NE close to Equator, indicating that the ITCZ was crossed during sampling. The West Pacific Region was characterised by weak winds from the South, while in the Central Pacific Region stronger winds were from the East. In summer, the East Pacific Region was characterised by moderate winds from NE and by weak southerly winds close to Central America. However, during winter season the winds were slightly stronger and were coming from NE. The Indian Region was influenced by the trade winds at the North and by the strong westerly winds at the South.

### Collection of airborne microbes

Air sampling was carried out using a commercially available cyclonic collector (*Coriolis-Δ*, Bertin Technologies^36^) placed at bow-side at ~10 m height over sea level at the top deck of the R/V *Hespérides*. The collector was operated using the procedures described earlier^2^. Briefly, air was drawn into the sampler at 300 l min^−1^ for 6 h (equivalent to 108 m^3^), and the collection liquid was refilled continuously by the system at a rate sufficient to match the loss of liquid by evaporation and re-aerosolisation measured during a previous 10 min run. Daily, a field blank was collected by sampling air for 2 min (equivalent to 0.6 m^3^). Samples were processed immediately after collection and the volume was adjusted to 15 ml with additional collection liquid if necessary.

### Microbial abundance and biomass

Prokaryotes and unicellular eukaryotes were counted in 5 ml aliquots of the air samples by epifluorescence microscopy (*Leika* DM 1000) following the procedures outlined in Mayol et al.^2^. The fraction of protists from total eukaryotic counts was determined using the blue excitation light at 480 nm, which allows the detection of chlorophyll pigments as a red-colour particle in autotrophic protists. Heterotrophic protists were expected to be in similar quantities than those of autotrophic protists, assuming that they are in a similar proportion in seawater. Thus, fungal spores were expected to be the remaining fraction. A minimum of 150 prokaryotes and unicellular eukaryotes were counted per sample in at least 12 different fields, or a minimum of 150 fields were counted for samples with lower abundances. Microbial losses by re-aerosolisation were corrected as described earlier^2^.

Additionally, we determined bacterial abundance in surface seawater by flow cytometry of SYBRGreen I stained samples^37^ to be used in the calculation of spray fluxes. The seawater was collected from the first metres (<5 m depth) of the mixed layer with Niskin bottles mounted on a rosette sampling system.

Microbial abundances were converted into C biomass using standard conversion factors of 12.4 fg C cell^−1^ for prokaryotes^38^ and 13 pg C cell^−1^ for fungal spores^39^.

### Microbial load

The microbial load in the boundary layer was estimated as the product between the measured microbial abundances in the air and the height of the ABL obtained from the Analysis of the European Center for Medium-range Weather Forecasts (ERA-Interim^40^^,^^41^) assuming that well-mixed conditions prevail^42^.

### Microbial identification by sequencing

The remaining 10 ml of the air samples were filtered through a 0.22 µm pore-size polycarbonate filter and stored at −80 °C. DNA was extracted from filters using a commercial kit (UltraClean® Soil DNA Isolation Kit, Mobio). In order to optimise the DNA yield, the filters were previously homogenised in a bead homogenizer (Precellys, Bertin Technologies) using autoclaved glass beads (0.5 mm) for 30 s at 2500 r.p.m. Nucleic acid extracts were stored at −20 °C until amplification. The V4 region of the 16S rDNA gene was amplified by PCR using primers 515 F (5′-GTGCCAGCMGCCGCGGTAA-3′) and 806 R (5′-GGACTACHVGGGTWTCTAAT-3′) suitable for amplification of most bacteria and archaea^43, 44^, generating ~250 bp amplicons suitable for community analysis^45^. The forward primer (515 F) contained a 454-B pyrosequencing adapter with a GA linker while the reverse primer (806 R) was linked by a AC linker to a unique 12 bp barcode sequence identifying each sample followed by a AG linker and a Roche 454-A sequencing adapter. Each 20 µl PCR reaction contained 5 µl of template DNA plus (final concentrations), 10 mM of dNTPs mixture, 5 µM of each primer and 0.01 units of Taq Polymerase (Takara) suspended in the buffer provided by the manufacturer. Additional negative control reactions (without DNA) were run with each batch of PCR reactions to check for possible contamination. The PCR consisted of an initial denaturing step at 94 °C for 3 min, 30 cycles of standard amplification (94 °C for 45 s, 50 °C for 60 s and 72 °C for 90 s) with a final elongation step of 72 °C for 10 min. The PCR products were checked by electrophoresis on 2% agarose gels. For each sample, we performed at least two independent PCR reactions and the products were pooled. The amplification products were cleaned and purified from dNTPs, primers and PCR reaction mix components using Ampure magnetic beads (Agencourt® AMPure® XP, Beckman Coulter). Purification and primer removal were checked by agarose electrophoresis. After purification and prior to sequencing, the DNA content of all purified PCR amplification products were quantified by using Quant-iT dsDNA Assay Kit (Invitrogen). The sequences generated were processed using the QIIME 1.8.0 software pipeline^46^ for quality control, demultiplexing according to the unique 12 bp barcodes, denoising, screening for chimeras and binning of phylotypes at a 97% similarity threshold. Only samples resulting in >800 quality controlled reads >200 bp were considered for the analysis. OTUs were assigned a phylogenetic affiliation by comparison with the SILVA database release 111^47^. We did not use the phylogenetic affiliation as an indication of terrestrial or marine origin because of the limited resolution afforded by short sequences for most OTUs was unlikely to provide unequivocal evidence of their origin for many of the OTUs. Instead, we used a more robust community-wide approach by comparing the whole airborne communities to a set of reference end-member communities from different sources. A reference database was constructed to represent a range of terrestrial and marine environments using surface seawater samples (<5 m depth) collected at 36 locations along the track of the Malaspina 2010 Circumnavigation Expedition plus additional 83 data sets using the same primer (Supplementary Table 4) from the SRA. This reference data set comprising a total of 119 samples was divided into two categories (terrestrial and marine) and used for attributing the relative contribution of marine and terrestrial sources to each of the airborne microbial assemblages using a community-wide approach by means of the software package ‘Source Tracker’^48^.

### Atmospheric parameters

Wind speed, air temperature, atmospheric pressure and humidity were measured in situ along the Malaspina 2010 Circumnavigation Expedition. These atmospheric parameters were extracted as 2-min average values from the continuous recording of the meteorological station installed on board the R/V *Hespérides*. In addition, volumetric measurements of precipitation were made from the rain collected in recipients placed on the top deck of the ship.

### Deposition flux

The dry (not associated to snow or rain conditions) deposition flux of airborne microorganisms (Fd, cells m^−2^ s^−1^) estimated for each sampled location was computed following Jurado et al.^17^ based on the formulation of Williams^49^ according to the equation

1 $${\rm{Fd}} = {v_{\rm{d}}} \times {C_{{\rm{air}}}}$$

where *v*_d_ is the deposition velocity (in m s^−1^) and *C*_air_ is the concentration of airborne microorganisms (cells m^−3^). The meteorological parameters needed to compute *v*_d_ (wind speed, temperature, humidity and pressure) were taken from the underway observations performed in the R/V *Hespérides* and averaged over the 6 h of sampling. The small sigma values of these averages (not shown) indicate that nearly steady-state conditions were present during sampling period at almost all the locations sampled. We assumed a density of 1.1 g cm^−3^ for biological particles^50^ and a mean diameter of 0.5 and 5 μm for prokaryotes and fungal spores respectively, as determined by epifluorescence microscopy.

### Spray flux

The sea-spray flux (Fs, cells m^−2^ s^−1^) estimated for all samples was computed using the microbial abundances measured in seawater as

2 $$F{\rm s} = {\dot V_{\rm{T}}} \times {C_{{\rm{water}}}}$$

where ${\dot V_{\rm{T}}}$ is the spray velocity estimated as the total volume flux of seawater spray into the atmosphere (m^3^ m^−2^ s^−1^) and *C*_water_ is the microbial abundance in sea surface water samples (cells m^−3^). As discussed in an earlier publication^2^, ${\dot V_{\rm{T}}}$ is computed using the formulations of Gong^51^ and considering a range of radii from 0.2 to 10 μm. Averaged values of the meteorological conditions (wind speed, temperature and humidity) registered by on board system of the R/V *Hespérides* during the 6 h of sampling were used in this calculation.

### Oceanic contribution to the pool of airborne microbes

In the absence of allochthonous inputs from land, the microbial abundance over oceanic location can be estimated as the result of the opposed fluxes of aerosolisation by spray formation and deposition. Thus, using the parameterisations of spray and deposition flux described above, the concentration of airborne microbes expected from local oceanic contributions can be estimated for the steady state where the deposition flux equals spray flux for the ambient conditions measured in situ. Following this methodology, the value of expected *C*_air_ was computed for each sample with the values measured of *C*_water_ and the averaged values of the meteorological magnitudes. The mixing processes related to the turbulence operate at time-scales shorter than our 6 h sampling period^52^. Therefore, well-mixed conditions are present and the dilution by turbulent motion is included in this balance. The departure between the observed and estimated *C*_air_ indicates the contribution of advective processes.

In a similar manner, we could constrain the range of global oceanic contributions because prokaryotic abundance at most locations in the surface ocean falls within a relatively narrow range from 5 × 10^5^ to 1.5 × 10^6^ prokaryotes ml^−1^ and the equilibrium depends mainly on the microbial abundances in surface waters and wind speed and to a lesser extent on the other meteorological variables used to calculate fluxes. Therefore, we estimated global oceanic contributions using standard conditions (*T* = 25 °C, RH = 80%, *P* = 1025 hPa) and a range of wind speeds and prokaryotic abundances encompassing the range commonly found in the ocean^53^ (Supplementary Fig. 9). This calculations assume non-preferential aerosolisation of microbes in the surface ocean, even if there is evidence indicating that microbes may be concentrated by bubbles and then aerosolisation rates may be higher than those expected from a homogeneous distribution microbes in the water column^54^ and some microbes could be even aerosolised more efficiently than others^55^. Thus, the potential contribution of marine microbes may be underestimated by this simple approach.

Additionally, the height of the ABL over land and ocean was computed as the average height of the NCEP/NCAR atmospheric reanalysis data of the potential temperature profiles corresponding to a potential temperature at least two degrees greater than the minimum temperature. Average values for the period between 1 January 1981 and 31 December 2010 were 817 ± 266 m (average ± SD) over the oceans and 564 ± 342 m over land similar to those reported by von Engel and Texeira^33^ for the period 1999–2009.

### Residence times

The residence time in the atmosphere for 50% of total sampled microorganisms in each location was calculated using the equation proposed by Mayol et al.^2^ that describes the remaining microbial load in the atmosphere as function of time and *v*_d_. To evaluate the effective distance that 50% of the particles can travel following the HYSPLIT^30^ forward trajectories of air masses at 10 m high, the maximum distance from the origin was considered during the respective residence times.

### Upscaling

The total load of microorganisms over the extension compressed between 40° S and 40° N was estimated by first determining the exponential fit between the observed prokaryotic and eukaryotic loads and their corresponding distances to the nearest land mass (Supplementary Fig. 10) and second by upscaling the loads to a regular grid of 1° × 1° covering the domain. The distances to the nearest land mass of each of the average positions of the ship during the 6 h of sampling and each of the grid points were obtained by using the GEBCO bathymetry of 1 × 1 min of spatial resolution (http://www.gebco.net). The estimated load at each grid point was multiplied by its corresponding cell grid area and finally added over the 40° S and 40° N region resulting in the total load value. Abundances of airborne prokaryotes m^−3^ and eukaryotes m^−3^ of air were upscaled at 1° × 1° resolution following the same procedure than for the estimated loads. The upscaled abundances were then interpolated to the NCEP/NCAR grid (2.5° × 2.5°) and used together with the 2011 annual mean of atmospheric fields from the NCEP/NCAR reanalysis (wind speed, relative humidity, air temperature and mean sea level pressure) to estimate the deposition fluxes encompassed between 40° S and 40° N. The spray fluxes over the study area were estimated by taking into account the minimum and maximum values of the observed abundances of prokaryotes on the sea surface water.

### Data availability

The measured abundances and calculated fluxes of microbes reported in this paper are included as Supplementary Information. The original sequences of airborne microbes reported in this study are available at the Sequence Read Archive (SRA, http://www.ncbi.nlm.nih.gov/sra/SRP074223) under Bioproject ID PRJNA319484 with accession numbers SAMN04903953 to SAMN04904051.

## Electronic supplementary material

Supplementary Information

## References

[1] Burrows SM, Elbert W, Lawrence MG, Pöschl U (2009). Bacteria in the global atmosphere–part 1: review and synthesis of literature data for different ecosystems. Atmos. Chem. Phys..

[2] Mayol E, Jiménez MA, Herndl GJ, Duarte CM, Arrieta JM (2014). Resolving the abundance and air-sea fluxes of airborne microorganisms in the North Atlantic Ocean. Front. Microbiol..

[3] Spracklen DV, Heald CL (2014). The contribution of fungal spores and bacteria to regional and global aerosol number and ice nucleation immersion freezing rates. Atmos. Chem. Phys..

[4] Burrows SM (2009). Bacteria in the global atmosphere–Part 2: Modeling of emissions and transport between different ecosystems. Atmos. Chem. Phys..

[5] O’Dowd CD, Leeuw Gde (2007). Marine aerosol production: a review of the current knowledge. Philos. Trans. R. Soc. Math. Phys. Eng. Sci..

[6] Morris CE (2014). Bioprecipitation: a feedback cycle linking Earth history, ecosystem dynamics and land use through biological ice nucleators in the atmosphere. Glob. Change Biol..

[7] Wilson TW (2015). A marine biogenic source of atmospheric ice-nucleating particles. Nature.

[8] Möhler O, DeMott PJ, Vali G, Levin Z (2007). Microbiology and atmospheric processes: the role of biological particles in cloud physics. Biogeosciences.

[9] Fröhlich-Nowoisky J (2012). Biogeography in the air: fungal diversity over land and oceans. Biogeosciences.

[10] Hervàs A, Camarero L, Reche I, Casamayor EO (2009). Viability and potential for immigration of airborne bacteria from Africa that reach high mountain lakes in Europe. Environ. Microbiol..

[11] Polymenakou PN (2012). Atmosphere: a source of pathogenic or beneficial microbes?. Atmosphere.

[12] Singh RP (2011). The emergence of Ug99 races of the stem rust fungus is a threat to world wheat production. Annu. Rev. Phytopathol..

[13] Matthias-Maser S, Brinkmann J, Schneider W (1999). The size distribution of marine atmospheric aerosol with regard to primary biological aerosol particles over the South Atlantic Ocean. Atmos. Environ..

[14] Cho BC, Hwang CY (2011). Prokaryotic abundance and 16S rRNA gene sequences detected in marine aerosols on the East Sea (Korea): prokaryotes above the East Sea. FEMS Microbiol. Ecol..

[15] DeLeon-Rodriguez N (2013). Microbiome of the upper troposphere: species composition and prevalence, effects of tropical storms, and atmospheric implications. Proc. Natl Acad. Sci. USA.

[16] Willeke K, Whitby KT (1975). Atmospheric aerosols: size distribution interpretation. J. Air Pollut. Control Assoc..

[17] Seinfeld, J. H. & Pandis, S. N. *Atmospheric Chemistry and Physics: From Air Pollution to Climate Change* (John Willey & Sons, 1998).

[18] Jurado E, Dachs J, Duarte CM, Simó R (2008). Atmospheric deposition of organic and black carbon to the global oceans. Atmos. Environ..

[19] Andreas EL (1998). A new sea spray generation function for wind speeds up to 32 m s^−1^. J. Phys. Oceanogr..

[20] Jurado E (2004). Atmospheric dry deposition of persistent organic pollutants to the atlantic and inferences for the global oceans. Environ. Sci. Technol..

[21] Kellogg CA, Griffin DW (2006). Aerobiology and the global transport of desert dust. Trends Ecol. Evol..

[22] NASA. Aerosol Optical Thickness (1 month – Aqua/MODIS). *Aerosol Optical Thickness* (*1 month – Aqua*/*MODIS*). Available at https://neo.sci.gsfc.nasa.gov/view.php?datasetId=MYDAL2_M_AER_OD&date=2011-05-09 (2011).

[23] Wu P-C, Tsai J-C, Li F-C, Lung S-C, Su H-J (2004). Increased levels of ambient fungal spores in Taiwan are associated with dust events from China. Atmos. Environ..

[24] Reponen T, Grinshpun SA, Conwell KL, Wiest J, Anderson M (2001). Aerodynamic versus physical size of spores: measurement and implication for respiratory deposition. Grana.

[25] Kunkel D (2012). Urban emission hot spots as sources for remote aerosol deposition: remote aerosol deposition. Geophys. Res. Lett..

[26] González-Gaya B, Zúñiga-Rival J, Ojeda M-J, Jiménez B, Dachs J (2014). Field measurements of the atmospheric dry deposition fluxes and velocities of polycyclic aromatic hydrocarbons to the global oceans. Environ. Sci. Technol..

[27] Ramana MV, Krishnan P, Nair SM, Kunhikrishnan PK (2004). Thermodynamic structure of the atmospheric boundary layer over the Arabian Sea and the Indian Ocean during pre-INDOEX and INDOEX-FFP campaigns. Ann. Geophys..

[28] Kirchman, D. L. *Processes in Microbial Ecology* (Oxford University Press, 2012).

[29] Jurado E (2005). Wet deposition of persistent organic pollutants to the global oceans. Environ. Sci. Technol..

[30] Stein AF (2015). NOAA’s HYSPLIT Atmospheric Transport and Dispersion Modeling System. Bull. Am. Meteorol. Soc..

[31] Després VR (2012). Primary biological aerosol particles in the atmosphere: a review. Tellus B.

[32] Archer CL, Jacobson MZ (2005). Evaluation of global wind power. J. Geophys. Res. Atmospheres.

[33] von Engeln A, Teixeira J (2013). A planetary boundary layer height climatology derived from ECMWF reanalysis data. J. Clim..

[34] Duarte CM (2015). Seafaring in the 21st century: the Malaspina 2010 Circumnavigation Expedition. Limnol. Oceanogr. Bull..

[35] Kistler R (2001). The NCEP–NCAR 50-year reanalysis: monthly means CD-ROM and documentation. Bull. Am. Meteorol. Soc..

[36] Carvalho E (2008). Performance of the Coriolis air sampler, a high-volume aerosol-collection system for quantification of airborne spores and pollen grains. Aerobiologia.

[37] Marie D, Partensky F, Jacquet S, Vaulot D (1997). Enumeration and cell cycle analysis of natural populations of marine picoplankton by flow cytometry using the nucleic acid stain SYBR Green I. Appl. Environ. Microbiol..

[38] Fukuda R, Ogawa H, Nagata T, Koike I (1998). Direct determination of carbon and nitrogen contents of natural bacterial assemblages in marine environments. Appl. Environ. Microbiol..

[39] Bauer H (2002). Determination of the carbon content of airborne fungal spores. Anal. Chem..

[40] Troen IB, Mahrt L (1986). A simple model of the atmospheric boundary layer; sensitivity to surface evaporation. Bound. Layer Meteorol..

[41] Dee DP (2011). The ERA-interim reanalysis: configuration and performance of the data assimilation system. Q. J. R. Meteorol. Soc..

[42] Wyngaard JC, Brost RA (1984). Top-down and bottom-up diffusion of a scalar in the convective boundary layer. J. Atmos. Sci..

[43] Bates ST (2011). Examining the global distribution of dominant archaeal populations in soil. ISME J..

[44] Bates ST, Cropsey GWG, Caporaso JG, Knight R, Fierer N (2011). Bacterial communities associated with the lichen symbiosis. Appl. Environ. Microbiol..

[45] Liu Z, Lozupone C, Hamady M, Bushman FD, Knight R (2007). Short pyrosequencing reads suffice for accurate microbial community analysis. Nucleic Acids Res..

[46] Caporaso JG (2010). QIIME allows analysis of high-throughput community sequencing data. Nat. Methods.

[47] Pruesse E (2007). SILVA: a comprehensive online resource for quality checked and aligned ribosomal RNA sequence data compatible with ARB. Nucleic Acids Res..

[48] Knights D (2011). Bayesian community-wide culture-independent microbial source tracking. Nat. Methods.

[49] Williams RM (1982). A model for the dry deposition of particles to natural water surfaces. Atmos. Environ. 1967.

[50] Bakken LR, Olsen RA (1983). Buoyant densities and dry-matter contents of microorganisms: conversion of a measured biovolume into biomass. Appl. Environ. Microbiol..

[51] Gong SL (2003). A parameterization of sea-salt aerosol source function for sub- and super-micron particles: sea-salt aerosol production. Glob. Biogeochem. Cycles.

[52] Lewis, E. R., Schwartz, S. E., Lewis, E. R. & Schwartz, S. E. *Sea Salt Aerosol Production: Mechanisms*, *Methods*, *Measurements and Models—A Critical Review* 9–99 (American Geophysical Union, 2004).

[53] Li WKW (1998). Annual average abundance of heterotrophic bacteria and Synechococcus in surface ocean waters. Limnol. Oceanogr..

[54] Blanchard DC, Syzdek LD (1982). Water-to-air transfer and enrichment of bacteria in drops from bursting bubbles. Appl. Environ. Microbiol..

[55] Fahlgren, C. *et al*. Seawater mesocosm experiments in the Arctic uncover differential transfer of marine bacteria to aerosols. *Environ*. *Microbiol*. *Rep*. **7**, 460–470 (2015). 10.1111/1758-2229.12273 25682947

