# Supplementary material for: Long-range transport of airborne microbes over the global tropical and subtropical ocean
Source: Nat Commun. 2017 Aug 4;8:201. doi: 10.1038/s41467-017-00110-9 (PMC5544686; doi:10.1038/s41467-017-00110-9)
Supplement: Supplementary file 1 — Supplementary Information [file 41467_2017_110_MOESM1_ESM.pdf]

File name: Supplementary Information
Description: Supplementary Figures, Supplementary Tables and Supplementary References

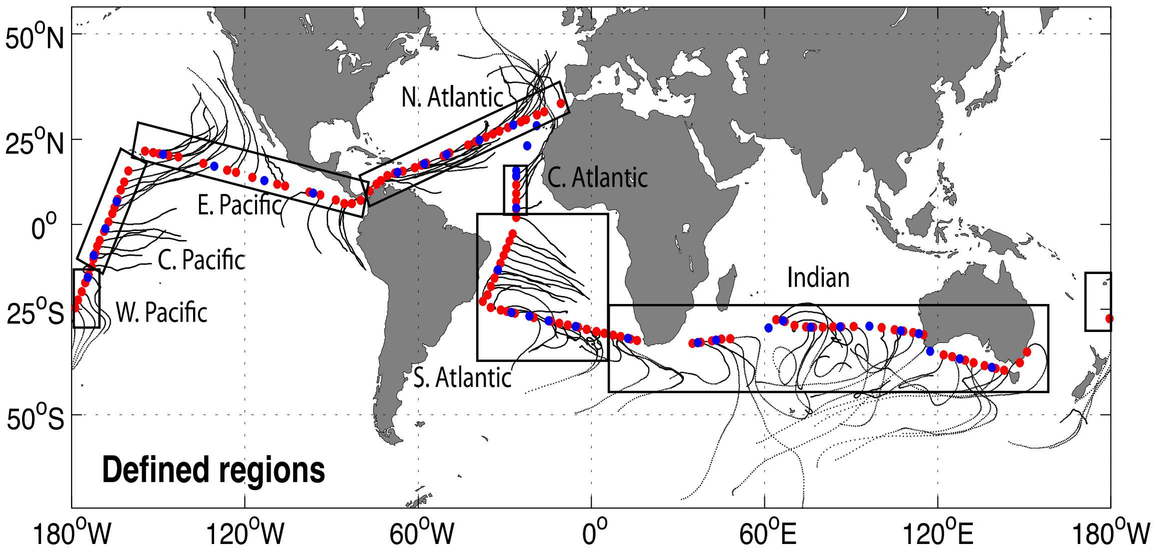

Supplementary Figure 1. Defined regions along the Malaspina 2010 Expedition and their respective sample groups. Black boxes show the seven groups of regions defined by their geographical origin as determined by the general atmospheric circulation patterns. The red dots represent the locations of the air samples and the black lines correspond to their respective backward trajectories. Blue dots represent the locations of surface seawater samples collected as reference for estimating the contribution of marine phylotypes.

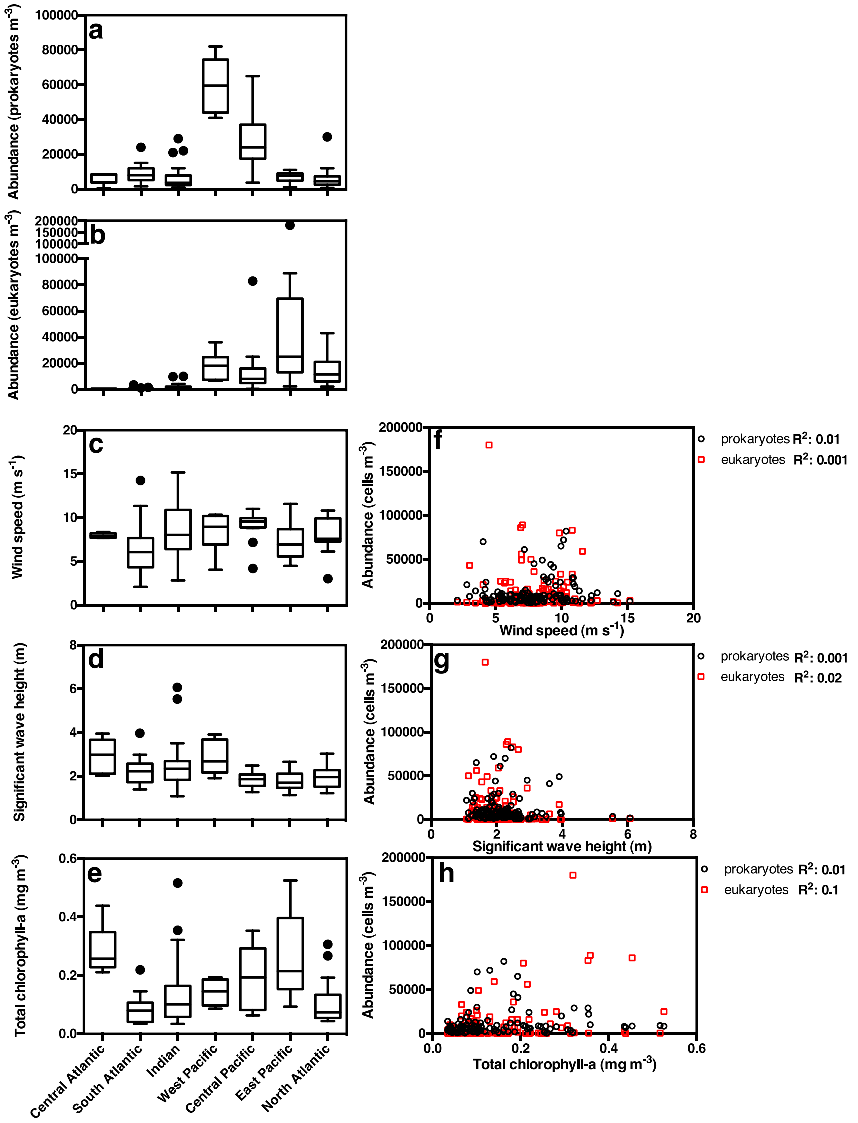

Supplementary Figure 2. Relationship between airborne microbial abundances and atmospheric and oceanic parameters. Panels (a) to (e) correspond to box plots showing the minimum, first quartile, median, third quartile and maximum values (black circles) of (a) prokaryotic abundances, (b) eukaryotic abundances, (c) measured wind speed, (d) significant wave height from the ERA-INTERIM reanalysis and, (e) Measured total chlorophyll-*a* for each defined region. Panels (f) to (h) correspond to correlations between prokaryotic and eukaryotic abundances and (f) measured wind speed, (g) significant wave height and, (h) Total chlorophyll-*a*. The R^2 values from correlations are reported in the graph.

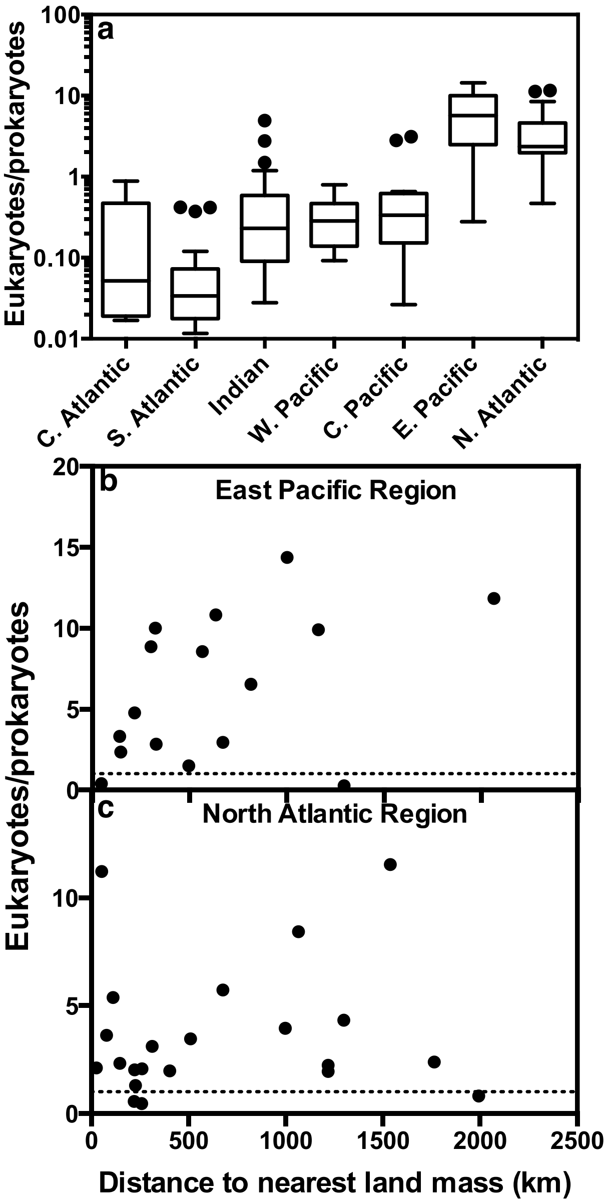

Supplementary Figure 3. The ratio between the abundance of eukaryotes and prokaryotes over the ocean. (a) Box plots show the distribution and median values of the ratio eukaryotes/prokaryotes in each defined region. Distance to nearest land mass versus the ratio between the abundance of eukaryotes and prokaryotes along (b) the East Pacific and (c) the North Atlantic region, both regions characterized by the presence of a large number of fungal spores. The dotted line shows the ratio = 1.

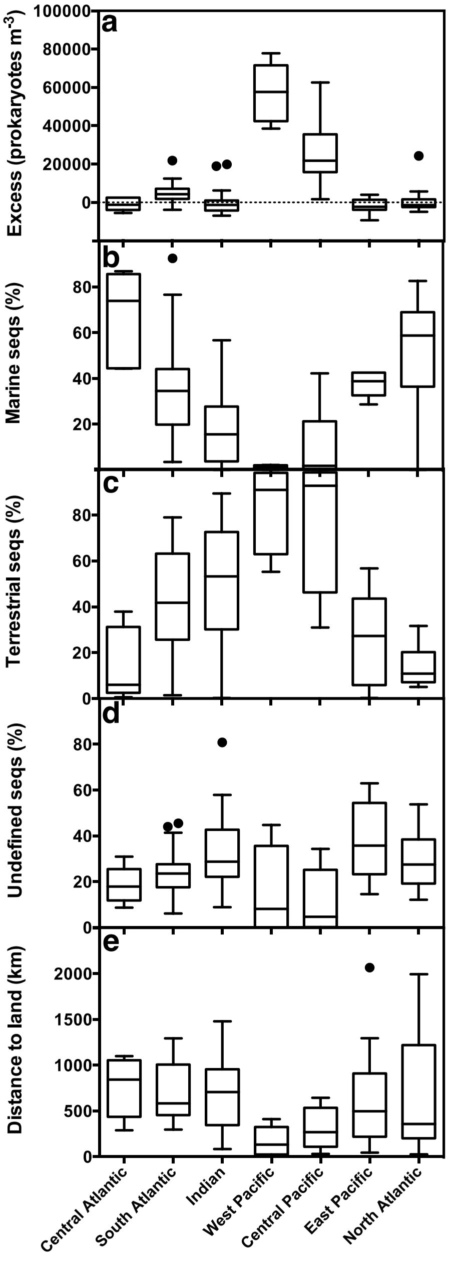

Supplementary Figure 4. Defined atmospheric region characterization. Panels correspond to box plots showing the minimum, first quartile, median, third quartile and maximum values (black circles) of (a) excess of prokaryotes, (b) relative contribution of marine sequences, (c) relative contribution of terrestrial sequences, (d) relative contribution of unknown sequences and; (e) distance to the nearest land mass for each defined region.

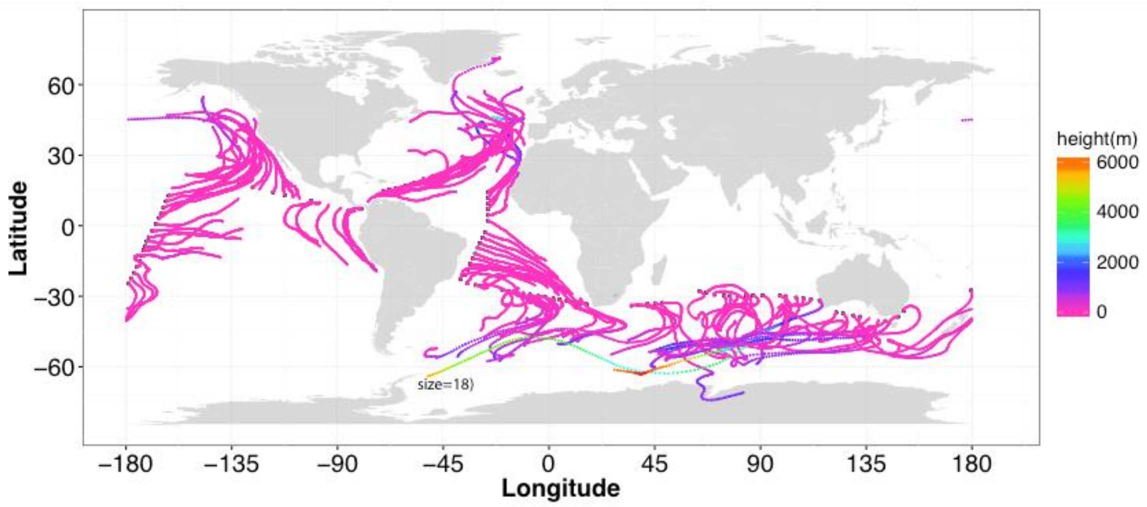

Supplemantary Figure 5. Three-dimensional representation of the computed retro-trajectories. The blue points at the end of each trace correspond to the sampling locations and lines correspond to the modelled back trajectories during the 7 days prior to sampling. The colour of the line represents the altitude modelled for the air mass sampled. The starting point of the model was set at 10 m over sea level for all the back trajectories.

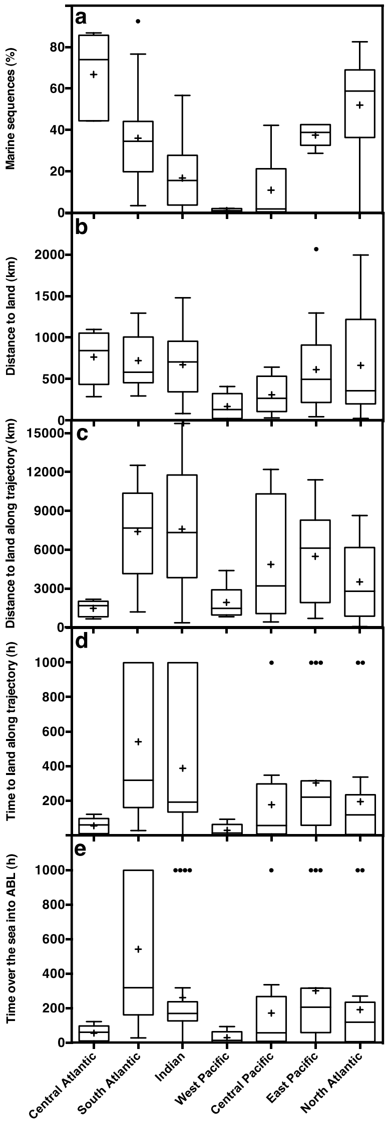

Supplementary Figure 6. Contribution of marine sequences associated to air trajectories in contact with sea or land. Panels (a) to (e) correspond to box plots showing the minimum, first quartile, median, average (+ symbol), third quartile and maximum values (black circles) of (a) relative contribution of marine sequences, (b) distance to the nearest land mass, (c) distance from 15 day air back trajectories calculated as the kilometres between the sampling site and the last point in contact with land, (d) hours equivalent to the time used to travel the distances in (c) and, (e) hours that the 15 day back trajectory remained over the ocean and into the ABL. Values = 999 were attributed to locations where the air sampled remained over ocean during the 15 days of the back trajectory.

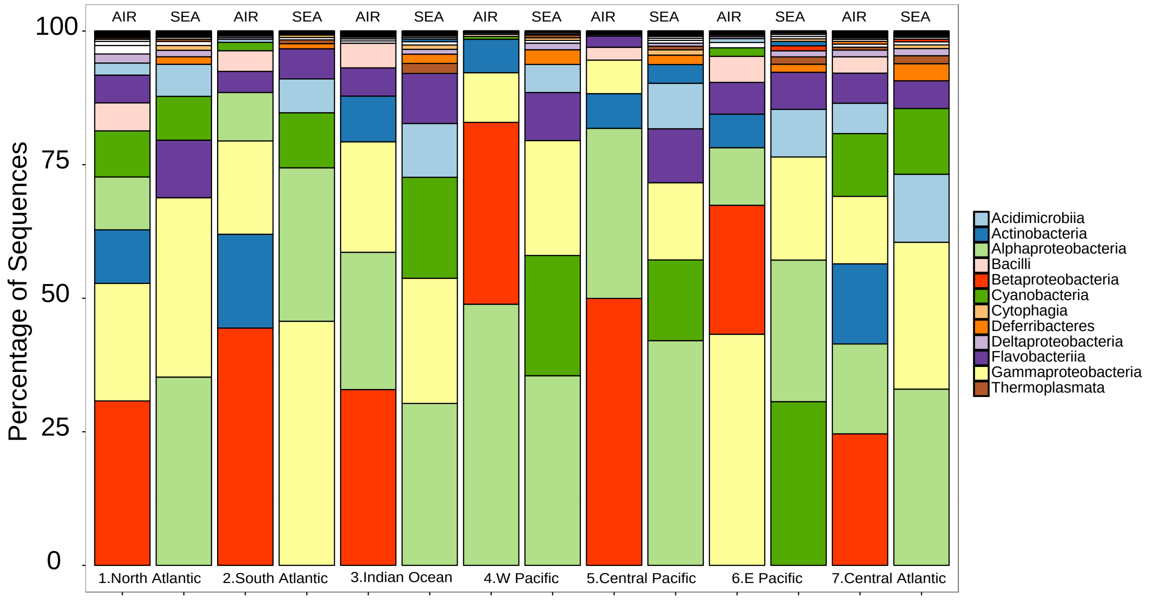

Supplementary Figure 7. Phylogenetic affiliation of the prokaryotic sequences obtained from the different air samples and reference seawater samples (<5m depth) grouped by region. Non-prokaryotic sequences classified as mitochondria or plastids were excluded from this figure.

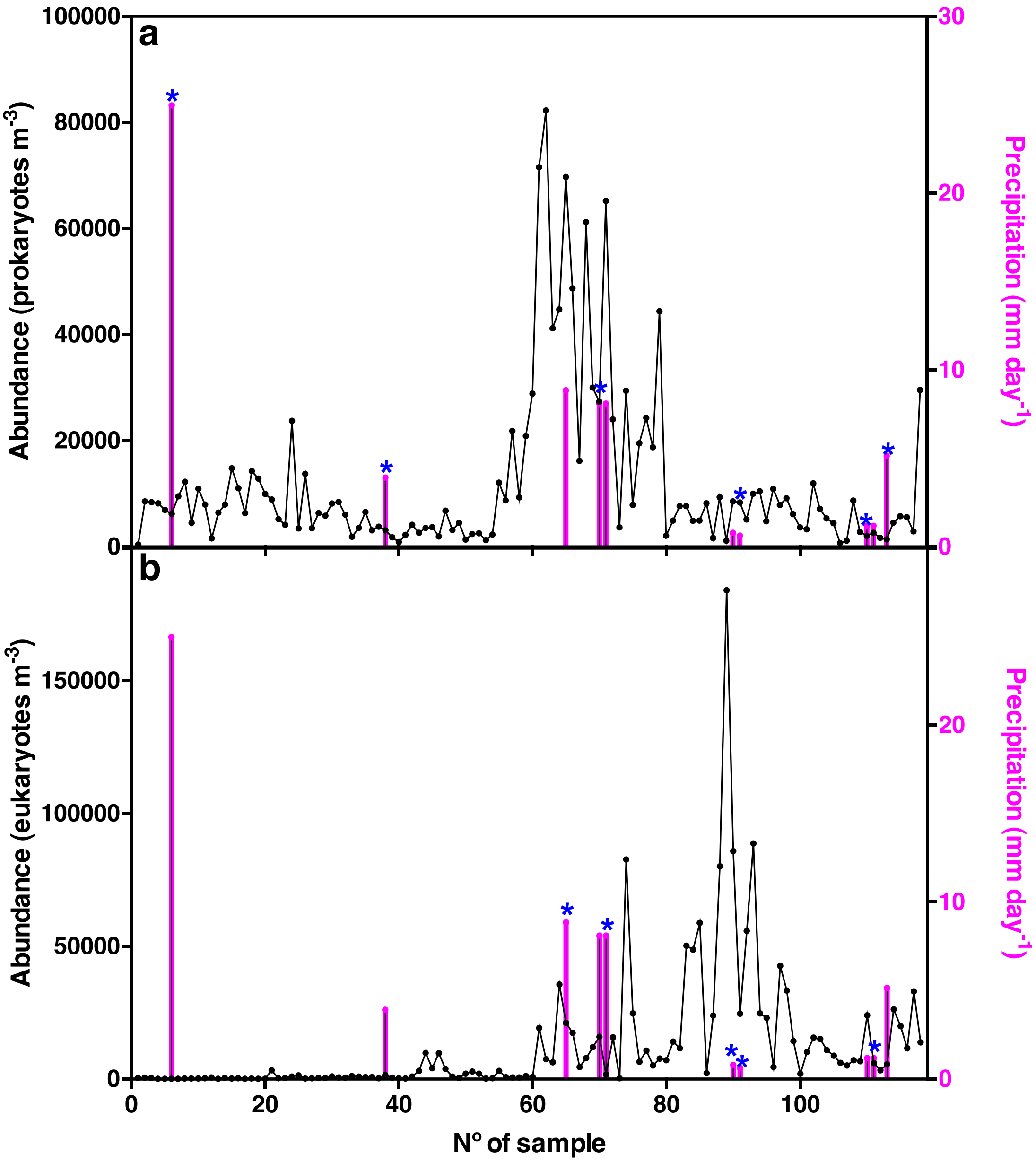

Supplementary Figure 8. Precipitation events associated to microbial abundances of air samples. (a) Abundance of prokaryotes and; (b) abundance of eukaryotes. The magenta bars represent the rain events associated to the 10 samples collected closest to the occurrence of the rainfall. Asterisks indicate the samples where microbial abundances were reduced as compared to the previous sample.

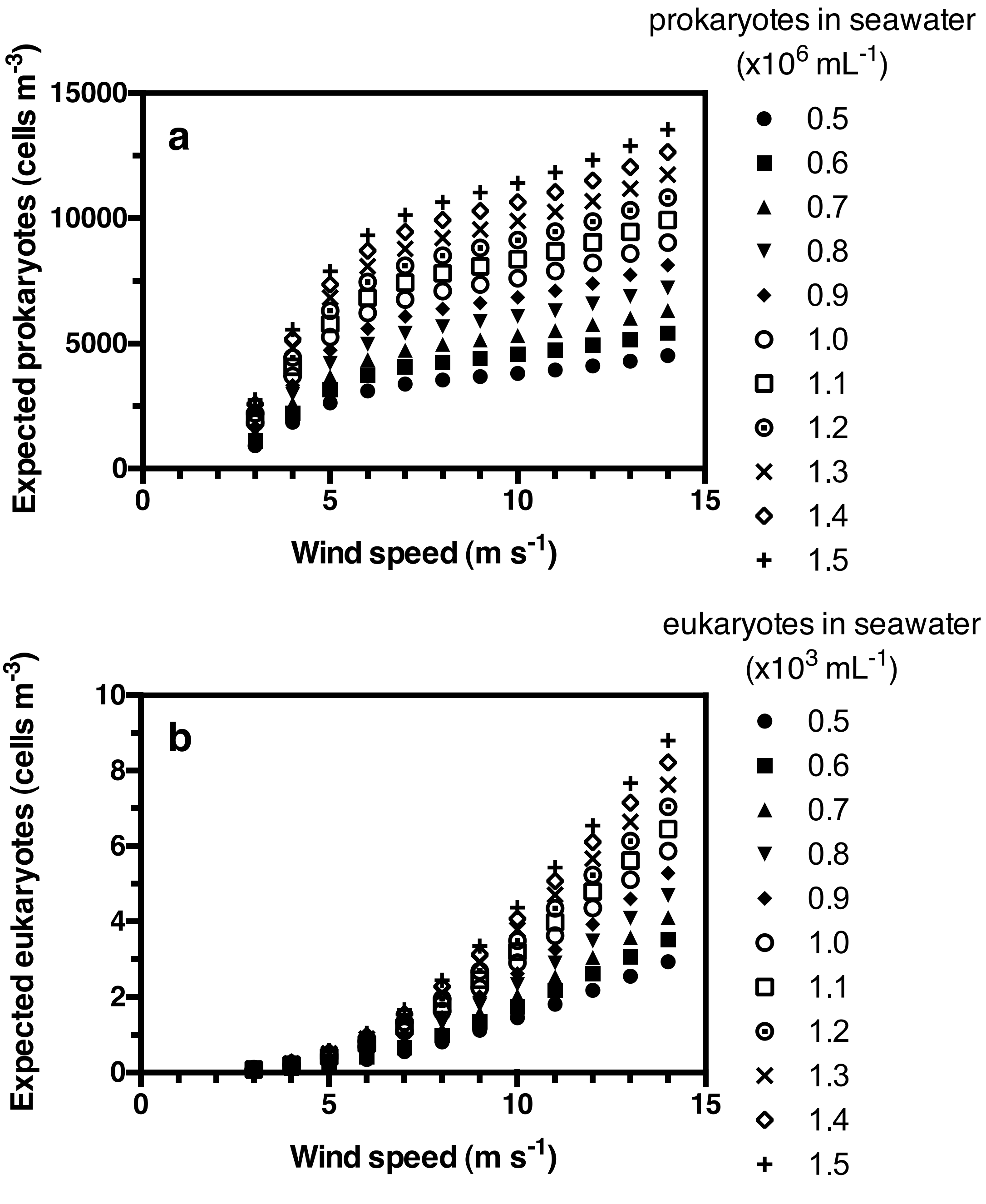

Supplementary Figure 9. Range of the expected local contribution of airborne prokaryotes and eukaryotes from the ocean. The range of (a) expected prokaryotes and (b) expected eukaryotes is composed by simulated values under conditions of spray and deposition fluxes at equilibrium, considering wind speeds between 3 and 14 m s^{-1} and surface abundances of seawater prokaryotes between 0.5×10^6 and 1.5×10^6 mL^{-1}.

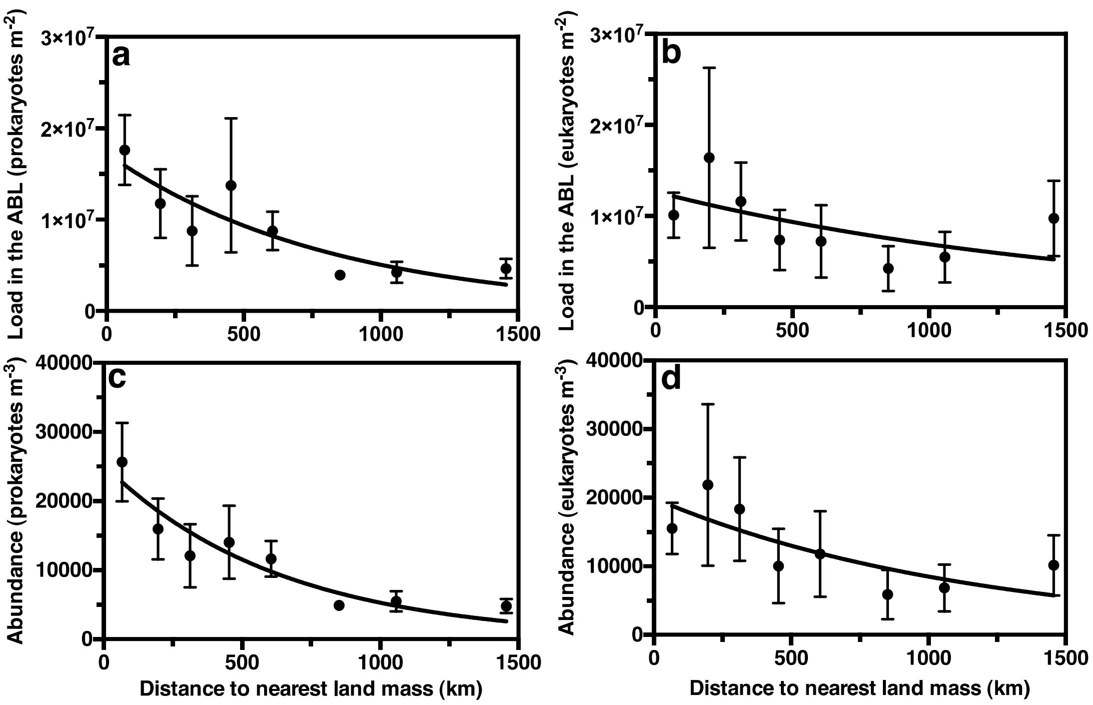

Supplementary Figure 10. Microbial loads and abundances versus distance to land. Exponential fits between the distance (D, km) to nearest land mass and (a) prokaryotic load, where y = $1.7\times10^7 e^{(-0.0012D)}$ and $R^2 = 0.78$, (b) eukaryotic load, where y = $1.3\times10^7 e^{(-0.00060D)}$ and $R^2 = 0.31$, (c) abundance of prokaryotes, where y = $25{,}217e^{(-0.0016D)}$ and $R^2 = 0.88$ and; (d) abundance of eukaryotes, where y = $19{,}883e^{(-0.00085D)}$ and $R^2 = 0.58$. Black dots represent the mean values and error bars represent the standard error of the mean (s.e.m). The solid lines show the fitted exponential models suggested by the Akaike information criterion (AIC).

Supplementary Table 1. Atmospheric, oceanic and biological data.

Sample	Region	Distance to coast (km)	SWH (m)	SWHw (m)	WSpeed ($m\ s^{-1}$)	sdWS ($m\ s^{-1}$)	Chl *a* ($mg\ m^{-3}$)	Rainfall ($mm\ d^{-1}$)	Prokaryotes Wet deposition ($cell\ m^{-2}\ d^{-1}$)	Prokaryotes Air abundance ($cell\ m^{-3}$)	Prokaryotes Water abundance ($cell\ ml^{-1}$)	Eukaryotes Wet deposition ($cell\ m^{-2}\ d^{-1}$)	Eukaryotes Air abundance ($cell\ m^{-3}$)	Eukaryotes Water abundance ($cell\ ml^{-1}$)
1	C. Atlantic	288	3	0	7.7	0.68	0.26			496	8.54E+05		436	854
2	C. Atlantic	586	2	0	7.68	0.93	0.26			8553	8.66E+05		528	866
3	C. Atlantic	843	2	1	8.08	0.92	0.21			8423	8.26E+05		438	826
4	C. Atlantic	1098	4	3	8.37	0.96	0.25			8231	1.32E+06		176	1315
5	C. Atlantic	1010	3	2	7.92	1.41	0.44			6955	1.32E+06		117	1316
6	S. Atlantic	850	3	1	7.83	0.99	0.22	25	7.2×10^{6}	6201	1.42E+06	0.14×10^{6}	117	1418
7	S. Atlantic	519	2	0	6.08	0.76	0.15			9521	1.60E+06		174	1601
8	S. Atlantic	560	2	0	7.05	1.16	0.11			12269	1.04E+06		204	1044
9	S. Atlantic	566	2	0	7.68	1.17	0.10			4537	9.53E+05		262	953
10	S. Atlantic	582	1	1	9.42	1.58	0.06			10989	6.70E+05		235	670
11	S. Atlantic	607	2	1	7.66	1.42	0.04			7991	6.02E+05		381	602
12	S. Atlantic	437	3	1	8.17	1.07	0.05			1609	5.33E+05		674	533
13	S. Atlantic	296	4	1	5.45	3.29	0.09			6452	4.93E+05		115	493
14	S. Atlantic	352	3	0	4.83	1.42	0.11			7989	5.50E+05		436	550
15	S. Atlantic	366	2	0	7.25	0.81	0.12			14815	6.81E+05		262	681
16	S. Atlantic	405	2	0	14.25	1.09				11099	6.62E+05		376	662
17	S. Atlantic	455	2	0	4.21	1.13	0.08			6398	5.25E+05		145	525
18	S. Atlantic	574	2	0	11.34	1.74	0.08			14296	4.63E+05		264	463
19	S. Atlantic	553	2	0	5.16	0.6	0.10			12862	4.72E+05		175	472
20	S. Atlantic	644	2	0	6.16	0.89	0.08			9979	5.01E+05		117	501
21	S. Atlantic	1171	2	0	4.02	1.39	0.09			8948	4.68E+05		3347	468
22	S. Atlantic	968	3	1	5.25	0.65	0.04			5204	5.58E+05		380	558
23	S. Atlantic	965	2	2	4.65	0.81	0.04			4155	3.38E+05		498	338
24	S. Atlantic	1006	3	2	4.26	0.52				23779	4.65E+05		901	465
25	S. Atlantic	1059	2	0	2.09	0.63	0.04			3457	5.91E+05		1437	591
26	S. Atlantic	1223	2	1	3.47	0.52	0.03			13774	4.96E+05		204	496
27	S. Atlantic	1294	2	2	4.32	1.91				3527	4.00E+05		386	400
28	S. Atlantic	1161	2	1	6.52	0.63	0.04			6360	7.97E+05		440	797
29	Indian O.	958	2	1	8.23	0.98	0.06			5852	7.02E+05		499	702
30	Indian O.	684	2	1	6.56	0.88	0.07			8186	8.09E+05		1081	809
31	Indian O.	347	3	1	5.41	0.81	0.06			8459	9.17E+05		673	917
32	Indian O.	139	3	1	7.51	1.23	0.22			6057	1.15E+06		557	1146
33	Indian O.	523	6	4	8.65	0.72	0.09			1902	1.17E+06		1143	1174
34	Indian O.	728	6	0	10.09	0.83	0.11			3563	1.17E+06		961	1174
35	Indian O.	947	3	0	8	1.26	0.11			6569	9.10E+05		731	910
36	Indian O.	834	3	1	12.26	1.26	0.14			3133	9.13E+05		729	913
37	Indian O.	846	4	0	12.22	0.85				3840	9.13E+05		323	913
38	Indian O.	900	2	1	5.57	2.8	0.09	4	0.05×10^{6}	3103	7.95E+05	0.02×10^{6}	1526	795
39	Indian O.	985	3	1	4.67	1.54	0.06			1841	6.77E+05		519	677

SWH: total significant wave height obtained from the ERA-INTERIM reanalysis.
SWHw: wind component of significant wave height.
WSpeed: Mean wind speed observed during sampling.
sdWS: Standard deviation o the mean wind speed observed during sampling.
Chl *a*: Chlorophyll-*a* concentration in surface waters around the sampling area.
Geographical locations are reported in Supplementary Table 2. Significant wave height, wet deposition and Chlorophyll-*a* concentrations were obtained as outlined in Supplementary methods.

									Prokaryotes			Eukaryotes		
Sample	Region	Distance to coast (km)	SWH (m)	SWHw (m)	WSpeed (m s^{-1})	sdWS (m s^{-1})	Chl *a* (mg m^{-3})	Rainfall (mm d^{-1})	Wet deposition (cell m^{-2} d^{-1})	Air abundance (cell m^{-3})	Water abundance (cell ml^{-1})	Wet deposition (cell m^{-2} d^{-1})	Air abundance (cell m^{-3})	Water abundance (cell ml^{-1})
40	Indian O.	1189	3	1	7.69	1.3	0.06			905	6.77E+05		350	677
41	Indian O.	964	3	2	4.28	1.38	0.04			2278	7.27E+05		234	727
42	Indian O.	888	3	1	5.47	0.98	0.05			4153	5.95E+05		906	595
43	Indian O.	912	3	1	6.4	0.79	0.04			2685	6.66E+05		3137	666
44	Indian O.	1031	2	1	7.16	0.61	0.04			3578	6.28E+05		9858	628
45	Indian O.	1203	2	0	6.79	1.44	0.03			3726	6.48E+05		4181	648
46	Indian O.	1479	2	0	6.43	1.02	0.04			1968	4.95E+05		9702	495
47	Indian O.	1333	2	1	5.52	2.02	0.06			6848	1.01E+05		3828	101
48	Indian O.	919	2	0	6.87	0.97	0.10			3161	5.82E+05		930	582
49	Indian O.	652	2	1	8.43	1.41	0.16			4525	4.34E+05		493	434
50	Indian O.	386	3	0	13.92	0.76	0.10			1379	9.16E+05		2056	916
51	Indian O.	90	2	0	15.18	2.29	0.12			2428	9.62E+05		2881	962
52	Indian O.	289	2	1	11.06	0.92	0.11			2588	8.44E+05		2017	844
53	Indian O.	345	1	0	8.04	1.13	0.15			1289	9.20E+05		294	920
54	Indian O.	602	2	1	8.15	1.58	0.17			2323	8.75E+05		320	875
55	Indian O.	452	2	2	12.69	0.78	0.18			12137	1.31E+06		3138	1308
56	Indian O.	375	2	1	12.26	0.79	0.24			8764	1.74E+06		787	1741
57	Indian O.	240	1	0	10.9	0.83	0.35			21876	2.42E+06		610	2423
58	Indian O.	127	2	0	8.64	1.16	0.52			9309	1.87E+06		609	1867
59	Indian O.	84	2	0	2.82	0.64				20944	1.31E+06		1196	1312
60	Indian O.	83	2	0	10.89	1.74	0.32			28869	1.15E+06		845	1146
61	W. Pacific	295	2	0	10.15	0.81	0.13			71593	5.08E+05		19242	508
62	W. Pacific	411	2	0	10.34	1.22	0.16			82262	5.75E+05		7574	575
63	W. Pacific	191	4	2	9.38	0.54	0.19			41226	3.65E+05		6376	365
64	W. Pacific	30	3	2	7.91	0.98	0.18			44774	1.55E+05		35605	155
65	W. Pacific	10	2	1	4.05	1.93	0.10	9	23×10^{6}	69737	7.62E+04	9×10^{6}	21115	76
66	W. Pacific	73	4	2	8.54	0.65	0.09			48714	1.54E+05		17400	154
67	C. Pacific	60	2	1	4.2	1.21	0.06			16225	3.47E+05		4571	347
68	C. Pacific	214	2	0	7.17	1.22				61241	3.96E+05		7999	396
69	C. Pacific	31	2	0	9.23	2	0.10			30005	4.39E+05		12070	439
70	C. Pacific	267	2	0	8.81	1.19	0.18	8	101×10^{6}	27356	4.81E+05	58×10^{6}	15957	481
71	C. Pacific	122	1	1	9.95	0.62	0.19	8	240×10^{6}	65220	3.51E+05	6×10^{6}	1727	351
72	C. Pacific	229	1	0	8.98	1.56	0.22			24025	3.19E+05		15761	319
73	C. Pacific	544	2	0	9.97	1.45	0.31			3688	2.70E+05		337	270
74	C. Pacific	523	2	1	10.8	1.19	0.35			29430	2.51E+05		82777	251
75	C. Pacific	305	2	0	9.55	0.48	0.28			7920	4.16E+05		24710	416
76	C. Pacific	95	1	0	9.55	0.57	0.29			19541	3.66E+05		6544	366
77	C. Pacific	372	2	1	9.91	0.69	0.08			24337	3.43E+05		10742	343
78	C. Pacific	634	2	0	11.01	0.67	0.08			18777	2.38E+05		5143	238
79	C. Pacific	645	2	0	9.18	0.79				44449	1.33E+05		7714	133
80	E. Pacific	141	2	1	5.31	0.93	0.19			2142	9.16E+05		7110	916
81	E. Pacific	328	2	0	6.15	0.79	0.09			4975	9.25E+05		14154	925
82	E. Pacific	496	1	0	8.32	1.1	0.17			7680	8.75E+05		11613	875

SWH: total significant wave height obtained from the ERA-INTERIM reanalysis.
SWHw: wind component of significant wave height.
WSpeed: Mean wind speed observed during sampling.
sdWS: Standard deviation o the mean wind speed observed during sampling.
Chl *a*: Chlorophyll-*a* concentration in surface waters around the sampling area.
Geographical locations are reported in Supplementary Table 2. Significant wave height, wet deposition and Chlorophyll-*a* concentrations were obtained as outlined in Supplementary methods.

									Prokaryotes			Eukaryotes		
Sample	Region	Distance to coast (km)	SWH (m)	SWHw (m)	WSpeed (m s^{-1})	sdWS (m s^{-1})	Chl *a* (mg m^{-3})	Rainfall (mm d^{-1})	Wet deposition (cell m^{-2} d^{-1})	Air abundance (cell m^{-3})	Water abundance (cell ml^{-1})	Wet deposition (cell m^{-2} d^{-1})	Air abundance (cell m^{-3})	Water abundance (cell ml^{-1})
83	E. Pacific	815	1	1	7.67	0.81				7671	1.06E+06		50230	1059
84	E. Pacific	1163	2	1	6.96	0.57	0.10			4922	1.24E+06		48778	1244
85	E. Pacific	2065	2	1	11.58	1.42	0.14			4971	9.06E+05		58840	906
86	E. Pacific	1296	2	1	9.08	1.16	0.44			8249	5.99E+05		2291	599
87	E. Pacific	1002	1	1	10.43	0.79	0.25			1665	1.09E+06		23932	1089
88	E. Pacific	566	3	1	9.83	0.57	0.21			9367	1.06E+06		80128	1065
89	E. Pacific	221	2	1	4.49	1.25	0.32			1191	9.41E+05		184120	941
90	E. Pacific	325	2	1	6.91	1.23	0.45	0.8	56×10^{6}	8573	2.68E+06	560×10^{6}	85805	2683
91	E. Pacific	672	1	1	5.76	2.18	1	0.6	112×10^{6}	8354	2.09E+06	332×10^{6}	24681	2087
92	E. Pacific	635	1	1	6.95	0.73	0			5162	1.76E+06		55872	1756
93	E. Pacific	302	2	1	7.04	0.69	0			10018	1.81E+06		88733	1806
94	E. Pacific	147	2	1	5.37	1.36	-			10469	1.20E+06		24716	1199
95	E. Pacific	217	2	0	5.67	0.84	-			4829	1.20E+06		23068	1199
96	E. Pacific	46	2	0	5.47	0.69	-			10923	1.20E+06		4548	1199
97	N. Atlantic	111	2	1	3.03	2.04	-			7918	1.20E+06		42622	1199
98	N. Atlantic	79	2	1	9.93	1.02	-			9195	8.06E+05		33399	806
99	N. Atlantic	147	1	1	9.93	1.02	0			6157	8.06E+05		14317	806
100	N. Atlantic	220	1	1	10.44	1.1	0			3666	7.83E+05		2075	783
101	N. Atlantic	314	2	2	7.42	0.73	0			3305	9.32E+05		10296	932
102	N. Atlantic	227	2	2	7.42	0.73	0			11978	9.38E+05		15605	938
103	N. Atlantic	26	2	1	6.49	1.03	-			7191	1.02E+06		15200	1023
104	N. Atlantic	223	1	0	7.54	1.45	0			5364	1.11E+06		10913	1109
105	N. Atlantic	404	1	0	9.31	0.85	0			4484	8.10E+05		8874	810
106	N. Atlantic	1067	3	2	7.43	0.67	0			729	8.14E+05		6146	814
107	N. Atlantic	1300	3	2	6.52	0.63	0			1192	7.14E+05		5167	714
108	N. Atlantic	1994	2	2	7.39	0.8	0			8732	5.27E+05		7168	527
109	N. Atlantic	1765	2	2	6.92	0.74	0			2808	6.62E+05		6707	662
110	N. Atlantic	1538	2	2	6.13	0.75	0	1.2	1.7×10^{6}	2079	6.11E+05	20×10^{6}	24006	611
111	N. Atlantic	1219	2	2	7.6	0.96	0	1.2	2.2×10^{6}	2690	5.61E+05	4.9×10^{6}	6023	561
112	N. Atlantic	1219	2	2	7.6	0.96	0			1730	5.10E+05		3375	510
113	N. Atlantic	998	2	2	10.15	0.79	0	5.2	2.0×10^{6}	1419	5.32E+05	8.0×10^{6}	5624	532
114	N. Atlantic	677	3	2	8.67	0.53	0			4577	5.54E+05		26211	554
115	N. Atlantic	510	3	2	8.67	0.53	0			5756	6.46E+05		19946	646
116	N. Atlantic	261	2	2	10.38	0.98	0			5578	7.41E+05		11616	741
117	N. Atlantic	54	2	2	10.81	2.36	0			2944	7.32E+05		33024	732
118	N. Atlantic	259	1	1	8.63	1.12	-			29576	7.32E+05		13854	732

SWH: total significant wave height obtained from the ERA-INTERIM reanalysis.
SWHw: wind component of significant wave height.
WSpeed: Mean wind speed observed during sampling.
sdWS: Standard deviation o the mean wind speed observed during sampling.
Chl *a*: Chlorophyll-*a* concentration in surface waters around the sampling area.
Geographical locations are reported in Supplementary Table 2. Significant wave height, wet deposition and Chlorophyll-*a* concentrations were obtained as outlined in Supplementary methods.

Supplementary Table 2. Accession numbers, sampling location, number of high quality reads >200bp, percentage of reads related to fungal mitochondrial sequences or plastid sequences obtained in this study.

SRA accession number	Sample Name	Longitude	Latitude	Good quality reads	%Eukaryotic sequences		Source attribution		
					Mitochondrial	Chloroplasts	Marine	Terrestrial	unknown
SAMN04903953	MLSP.AIR.001	-26.001	14.370	1389	16%	1%	45%	25%	31%
SAMN04903954	MLSP.AIR.002	-25.997	11.926	1168	10%	1%	44%	38%	18%
SAMN04903955	MLSP.AIR.003	-25.998	9.306	817	6%	3%	74%	6%	20%
SAMN04903956	MLSP.AIR.004	-25.997	7.037	1253	1%	0%	87%	4%	9%
SAMN04903957	MLSP.AIR.005	-26.001	4.556	797	0%	1%	84%	1%	15%
SAMN04903958	MLSP.AIR.006	-26.002	2.100	1064	1%	0%	75%	6%	19%
SAMN04903959	MLSP.AIR.007	-27.270	-2.898	1646	0%	1%	12%	64%	24%
SAMN04903960	MLSP.AIR.008	-28.333	-5.170	1091	0%	1%	34%	43%	23%
SAMN04903961	MLSP.AIR.009	-29.375	-7.368	1522	0%	0%	28%	26%	45%
SAMN04903962	MLSP.AIR.010	-30.359	-9.463	978	0%	0%	20%	51%	29%
SAMN04903963	MLSP.AIR.011	-31.493	-11.848	1208	1%	1%	5%	70%	24%
SAMN04903964	MLSP.AIR.012	-32.526	-13.994	2191	1%	0%	41%	15%	44%
SAMN04903965	MLSP.AIR.013	-33.551	-16.133	1330	3%	0%	8%	67%	25%
SAMN04903966	MLSP.AIR.014	-34.727	-18.563	1371	1%	1%	21%	63%	16%
SAMN04903967	MLSP.AIR.015	-35.924	-20.975	1465	6%	0%	19%	68%	13%
SAMN04903968	MLSP.AIR.016	-37.620	-22.818	1627	1%	0%	27%	46%	28%
SAMN04903969	MLSP.AIR.017	-34.907	-24.546	1895	22%	1%	39%	36%	25%
SAMN04903970	MLSP.AIR.018	-31.314	-25.216	1697	17%	1%	39%	42%	19%
SAMN04903971	MLSP.AIR.019	-29.228	-25.583	1601	18%	0%	54%	27%	19%
SAMN04903972	MLSP.AIR.020	-26.491	-26.063	2181	0%	0%	92%	1%	6%
SAMN04903973	MLSP.AIR.021	-19.896	-27.236	1819	12%	0%	35%	49%	16%
SAMN04903974	MLSP.AIR.022	-14.062	-28.218	1653	26%	1%	44%	29%	27%
SAMN04903975	MLSP.AIR.023	-11.023	-28.770	1294	12%	0%	41%	17%	41%
SAMN04903976	MLSP.AIR.024	-8.144	-29.278	1989	2%	0%	77%	13%	10%
SAMN04903977	MLSP.AIR.025	-4.545	-29.880	1314	17%	0%	3%	79%	18%
SAMN04903978	MLSP.AIR.026	-1.593	-30.426	1550	10%	0%	31%	35%	34%
SAMN04903979	MLSP.AIR.027	1.797	-30.996	1807	54%	0%	36%	45%	20%
SAMN04903980	MLSP.AIR.028	4.527	-31.429	1411	8%	0%	47%	26%	28%
SAMN04903981	MLSP.AIR.029	7.537	-31.935	1541	21%	41%	16%	40%	44%
SAMN04903982	MLSP.AIR.030	10.149	-32.325	1699	7%	1%	37%	39%	25%
SAMN04903983	MLSP.AIR.031	13.630	-32.953	1608	13%	2%	27%	30%	43%
SAMN04903984	MLSP.AIR.032	15.913	-33.337	2585	11%	1%	29%	25%	46%
SAMN04903985	MLSP.AIR.033	35.126	-34.039	2139	1%	1%	34%	30%	36%
SAMN04903986	MLSP.AIR.034	37.926	-33.749	3757	14%	1%	30%	30%	40%
SAMN04903987	MLSP.AIR.035	41.868	-33.345	2606	0%	1%	24%	26%	50%
SAMN04903988	MLSP.AIR.036	45.090	-32.973	2115	4%	6%	4%	63%	33%

The source attribution columns refer to the % of the sample attributed to marine or terrestrial sources following the Source Tracker partitioning procedure. The sequences were deposited in the Sequence Read Archive http://www.ncbi.nlm.nih.gov/sra under bioproject ID PRJNA3194.

SRA accession number	Sample Name	Longitude	Latitude	Good quality reads	%Eukaryotic sequences		Source attribution		
					Mitochondrial	Chloroplasts	Marine	Terrestrial	unknown
SAMN04903989	MLSP.AIR.037	48.151	-32.842	2711	10%	0%	17%	65%	18%
SAMN04903990	MLSP.AIR.038	64.134	-27.856	2262	0%	1%	19%	41%	40%
SAMN04903991	MLSP.AIR.039	67.021	-28.335	2010	3%	0%	16%	57%	27%
SAMN04903992	MLSP.AIR.040	70.369	-29.350	2240	5%	0%	9%	60%	31%
SAMN04903993	MLSP.AIR.041	74.459	-29.786	2383	3%	0%	15%	51%	34%
SAMN04903994	MLSP.AIR.042	76.949	-29.891	2562	2%	0%	3%	77%	20%
SAMN04903995	MLSP.AIR.043	80.195	-29.815	2734	6%	1%	3%	72%	25%
SAMN04903996	MLSP.AIR.044	83.828	-29.784	2043	3%	0%	20%	57%	23%
SAMN04903997	MLSP.AIR.045	86.697	-29.736	2650	1%	1%	15%	46%	39%
SAMN04903998	MLSP.AIR.046	90.942	-29.677	2233	5%	0%	0%	75%	25%
SAMN04903999	MLSP.AIR.047	100.599	-29.983	2430	9%	0%	1%	73%	26%
SAMN04904000	MLSP.AIR.048	105.137	-30.547	2057	2%	0%	1%	79%	21%
SAMN04904001	MLSP.AIR.049	108.154	-30.918	2064	6%	0%	4%	85%	11%
SAMN04904002	MLSP.AIR.050	111.507	-31.283	2062	0%	2%	57%	0%	43%
SAMN04904003	MLSP.AIR.051	115.092	-31.788	1647	6%	0%	15%	60%	25%
SAMN04904004	MLSP.AIR.052	122.172	-36.870	1947	5%	0%	2%	89%	9%
SAMN04904005	MLSP.AIR.053	125.070	-37.309	1653	10%	0%	32%	47%	20%
SAMN04904006	MLSP.AIR.054	129.556	-38.202	1193	1%	1%	33%	10%	58%
SAMN04904007	MLSP.AIR.055	132.458	-38.729	1241	0%	0%	8%	79%	13%
SAMN04904008	MLSP.AIR.058	143.119	-40.519	1087	3%	0%	13%	36%	51%
SAMN04904009	MLSP.AIR.059	148.536	-38.738	1390	0%	0%	0%	19%	81%
SAMN04904010	MLSP.AIR.060	151.078	-36.153	2365	5%	1%	22%	56%	23%
SAMN04904011	MLSP.AIR.061	179.608	-27.527	1396	34%	0%	2%	65%	33%
SAMN04904012	MLSP.AIR.062	-178.894	-24.613	2610	26%	0%	1%	87%	12%
SAMN04904013	MLSP.AIR.063	-177.889	-22.389	2309	1%	0%	0%	55%	45%
SAMN04904014	MLSP.AIR.064	-176.578	-20.054	2566	56%	0%	2%	98%	0%
SAMN04904015	MLSP.AIR.065	-175.317	-17.512	1520	44%	0%	0%	100%	0%
SAMN04904016	MLSP.AIR.066	-174.150	-15.174	2158	30%	0%	1%	95%	4%
SAMN04904017	MLSP.AIR.067	-173.114	-12.878	2287	12%	1%	23%	42%	34%
SAMN04904018	MLSP.AIR.068	-172.526	-10.478	1548	13%	0%	0%	96%	4%
SAMN04904019	MLSP.AIR.069	-171.903	-8.828	2350	5%	1%	0%	100%	0%
SAMN04904020	MLSP.AIR.070	-171.271	-6.626	1628	18%	0%	0%	100%	0%
SAMN04904021	MLSP.AIR.071	-170.318	-4.915	2825	11%	0%	1%	99%	1%
SAMN04904022	MLSP.AIR.072	-168.814	-2.148	2487	5%	0%	15%	58%	27%
SAMN04904023	MLSP.AIR.073	-167.277	0.799	2237	20%	0%	0%	90%	11%
SAMN04904024	MLSP.AIR.074	-165.983	3.311	969	0%	1%	42%	31%	27%
SAMN04904025	MLSP.AIR.075	-165.166	5.165	2453	7%	2%	41%	39%	20%
SAMN04904026	MLSP.AIR.076	-164.099	7.629	2191	15%	0%	2%	98%	0%
SAMN04904027	MLSP.AIR.077	-163.025	10.373	1740	9%	0%	2%	98%	0%
SAMN04904028	MLSP.AIR.078	-161.858	12.725	1692	25%	0%	5%	90%	6%

The source attribution columns refer to the % of the sample attributed to marine or terrestrial sources following the Source Tracker partitioning procedure. The sequences were deposited in the Sequence Read Archive http://www.ncbi.nlm.nih.gov/sra under bioproject ID PRJNA3194.

SRA accession number	Sample Name	Longitude	Latitude	Good quality reads	%Eukaryotic sequences		Source attribution		
					Mitochondrial	Chloroplasts	Marine	Terrestrial	unknown
SAMN04904029	MLSP.AIR.086	-126.115	16.284	2868	73%	0%	34%	30%	36%
SAMN04904030	MLSP.AIR.088	-117.410	14.191	1839	74%	0%	37%	0%	63%
SAMN04904031	MLSP.AIR.094	-85.589	6.278	1291	48%	0%	29%	57%	15%
SAMN04904032	MLSP.AIR.095	-82.972	6.352	854	36%	0%	41%	27%	32%
SAMN04904033	MLSP.AIR.096	-79.867	7.262	1259	21%	0%	43%	12%	46%
SAMN04904034	MLSP.AIR.099	-72.962	13.204	1102	35%	1%	66%	9%	25%
SAMN04904035	MLSP.AIR.100	-70.583	14.640	1695	57%	0%	62%	13%	24%
SAMN04904036	MLSP.AIR.101	-68.004	15.363	1291	58%	0%	76%	9%	15%
SAMN04904037	MLSP.AIR.102	-65.271	15.988	1100	47%	2%	55%	16%	29%
SAMN04904038	MLSP.AIR.103	-61.095	16.997	988	56%	0%	52%	20%	28%
SAMN04904039	MLSP.AIR.104	-58.854	17.739	847	52%	0%	18%	32%	50%
SAMN04904040	MLSP.AIR.105	-57.258	18.307	1058	15%	0%	35%	27%	38%
SAMN04904041	MLSP.AIR.107	-49.140	21.164	893	46%	1%	83%	5%	12%
SAMN04904042	MLSP.AIR.110	-36.611	25.615	1193	61%	1%	70%	11%	19%
SAMN04904043	MLSP.AIR.113	-28.895	28.237	949	36%	0%	41%	5%	54%
SAMN04904044	MLSP.AIR.116	-18.824	31.574	1198	50%	0%	65%	7%	27%
SAMN04904045	MLSP.AIR.XX2	-17.246	-27.701	2134	2%	0%	42%	19%	39%
SAMN04904046	MLSP.AIR.XX3	-53.475	19.765	1153	0%	0%	62%	9%	29%
SAMN04904047	MLSP.AIR.XX4	-138.082	19.088	2198	69%	0%	53%	6%	41%
SAMN04904048	MLSP.AIR.XX5	-131.904	17.684	3024	56%	0%	75%	1%	24%
SAMN04904049	MLSP.AIR.XX6	-112.529	13.010	2955	16%	0%	68%	2%	30%
SAMN04904050	MLSP.AIR.XX7	-101.297	10.527	1586	67%	0%	73%	1%	26%
SAMN04904051	MLSP.AIR.XX8	98.194	-29.703	2066	14%	0%	2%	89%	9%

The source attribution columns refer to the % of the sample attributed to marine or terrestrial sources following the Source Tracker partitioning procedure. The sequences were deposited in the Sequence Read Archive http://www.ncbi.nlm.nih.gov/sra under bioproject ID PRJNA3194.

Supplementary Table 3. Air-sea exchange fluxes and distance travelled by microbes.

	Prokaryotes			Eukaryotes		
	**Spray** (cells m^{-2} d^{-1})	**Dry deposition** (cells m^{-2} d^{-1})	**Distance** (km)	**Spray** (cells m^{-2} d^{-1})	**Dry deposition** (cells m^{-2} d^{-1})	**Distance** (km)
Central Atlantic	1.9×10^5-3.9×10^5 (2.2×10^5)	1.7×10^4-3.6×10^5 (2.9×10^5)	1.4×10^3-3.8×10^3 (3.4×10^3)	1.9×10^2-3.9×10^2 (2.2×10^2)	1.7×10^4-7.3×10^4 (6.0×10^4)	1.4×10^3-2.9×10^3 (2.7×10^3)
South Atlantic	1.6×10^3-1.2×10^6 (5.3×10^4)	3×10^4-2.2×10^6 (1.2×10^5)	1.2×10^3-9.1×10^4 (2.5×10^4)	1.6×10^0-1.2×10^3 (5.3×10^1)	1.4×10^4-4×10^5 (4.6×10^4)	9.9×10^2-6.2×10^4 (6.0×10^3)
Indian Ocean	7.3×10^3-2.2×10^6 (2.2×10^5)	1.6×10^4-2.7×10^6 (1.5×10^5)	4.9×10^2-7.4×10^4 (7.2×10^3)	7.3×10^0-2.2×10^3 (2.2×10^2)	2.8×10^4-1.4×10^6 (1.5×10^5)	4.9×10^2-6×10^4 (3.4×10^3)
West Pacific	1.9×10^3-3.5×10^5 (1.0×10^5)	6×10^5-6.4×10^6 (2.3×10^6)	3.1×10^4-7×10^4 (3.9×10^4)	1.9×10^0-3.5×10^2 (1.0×10^2)	1×10^6-5.2×10^6 (2.6×10^6)	9×10^3-5.8×10^4 (3.9×10^4)
Central Pacific	9.9×10^3-1.9×10^5 (1.7×10^5)	1.4×10^5-4.5×10^6 (1.6×10^6)	1.7×10^4-6.3×10^4 (3.1×10^4)	9.9×10^0-1.9×10^2 (1.7×10^2)	6.7×10^4-1.7×10^7 (1.3×10^6)	1.6×10^3-5.3×10^4 (2.9×10^4)
East Pacific	3.4×10^4-8.2×10^5 (2.3×10^5)	1×10^4-6.5×10^5 (1.3×10^5)	3.6×10^2-9.7×10^4 (3.4×10^3)	3.4×10^1-8.2×10^2 (2.3×10^2)	3.8×10^5-2.2×10^7 (4.5×10^6)	2×10^2-5.3×10^4 (1.9×10^3)
North Atlantic	1.1×10^4-5.2×10^5 (1.9×10^5)	1.9×10^4-1.3×10^6 (1.7×10^5)	2.9×10^2-5.6×10^4 (4.7×10^3)	1.1×10^1-5.2×10^2 (1.9×10^2)	3.9×10^5-6.6×10^6 (1.8×10^6)	2.9×10^2-2.2×10^4 (3.3×10^3)
**Total**	**1.6×10^3-2.2×10^6 (1.7×10^5)**	**1×10^4-6.4×10^6 (2.1×10^5)**	**2.9×10^2-9.7×10^4 (2.2×10^4)**	**1.6×10^0 -2.2×10^3 (1.7×10^2)**	**1.4×10^4-2.2×10^7 (5.1×10^5)**	**2×10^2-6.2×10^4 (5.8×10^3)**

Range and median (in brackets) for spray, dry deposition and distance travelled by prokaryotes and eukaryotes (divided by regions and total values) along the Malaspina 2010 Circumnavigation Expedition. Distances showed in the table were calculated for remaining microbial load of 50%.

Supplementary Table 4. Reference samples used to trace the source of the airborne microbial assemblages obtained from the NCBI SRA repository (http://www.ncbi.nlm.nih.gov/sra)

Accession number	Source	Category
**ERR440975**	Continental air	Terrestrial
**ERR440935**	Continental air	Terrestrial
**ERR440918**	Continental air	Terrestrial
**ERR440906**	Continental air	Terrestrial
**ERR440897**	Continental air	Terrestrial
**ERR440895**	Continental air	Terrestrial
**ERR440870**	Continental air	Terrestrial
**ERR440837**	Continental air	Terrestrial
**ERR440985**	Freshwater lake	Terrestrial
**ERR440980**	Freshwater lake	Terrestrial
**ERR440941**	Freshwater lake	Terrestrial
**ERR440913**	Freshwater lake	Terrestrial
**ERR440880**	Freshwater lake	Terrestrial
**ERR440871**	Freshwater lake	Terrestrial
**ERR440823**	Freshwater lake	Terrestrial
**ERR440813**	Freshwater lake	Terrestrial
**ERR440942**	Freshwater lake	Terrestrial
**ERR440931**	leaf litter	Terrestrial
**ERR440917**	leaf litter	Terrestrial
**ERR440889**	leaf litter	Terrestrial
**ERR440877**	leaf litter	Terrestrial
**ERR440875**	leaf litter	Terrestrial
**ERR440866**	leaf litter	Terrestrial
**ERR440844**	leaf litter	Terrestrial
**ERR440827**	leaf litter	Terrestrial
**ERR440809**	leaf litter	Terrestrial
**ERR440872**	Lichen	Terrestrial
**ERR440859**	Lichen	Terrestrial
**ERR440839**	Lichen	Terrestrial
**ERR440836**	Lichen	Terrestrial
**ERR440817**	Lichen	Terrestrial
**ERR440986**	soil	Terrestrial
**ERR440978**	soil	Terrestrial
**ERR440970**	soil	Terrestrial
**ERR440969**	soil	Terrestrial
**ERR440967**	soil	Terrestrial
**ERR440966**	soil	Terrestrial
**ERR440950**	soil	Terrestrial

Accession number	Source	Category
**ERR440947**	soil	Terrestrial
**ERR440946**	soil	Terrestrial
**ERR440944**	soil	Terrestrial
**ERR440939**	soil	Terrestrial
**ERR440934**	soil	Terrestrial
**ERR440929**	soil	Terrestrial
**ERR440927**	soil	Terrestrial
**ERR440926**	soil	Terrestrial
**ERR440925**	soil	Terrestrial
**ERR440924**	soil	Terrestrial
**ERR440921**	soil	Terrestrial
**ERR440900**	soil	Terrestrial
**ERR440898**	soil	Terrestrial
**ERR440894**	soil	Terrestrial
**ERR440883**	soil	Terrestrial
**ERR440882**	soil	Terrestrial
**ERR440874**	soil	Terrestrial
**ERR440868**	soil	Terrestrial
**ERR440864**	soil	Terrestrial
**ERR440860**	soil	Terrestrial
**ERR440857**	soil	Terrestrial
**ERR440854**	soil	Terrestrial
**ERR440853**	soil	Terrestrial
**ERR440850**	soil	Terrestrial
**ERR440848**	soil	Terrestrial
**ERR440845**	soil	Terrestrial
**ERR440824**	soil	Terrestrial
**ERR440821**	soil	Terrestrial
**ERR440820**	soil	Terrestrial
**ERR440811**	soil	Terrestrial
**ERR440807**	soil	Terrestrial
**ERR440797**	soil	Terrestrial
**ERR440796**	soil	Terrestrial
**ERR440795**	soil	Terrestrial
**MLSPN.WC.0026**	surface open ocean (This study)	Marine
**MLSPN.WC.0036**	surface open ocean (This study)	Marine
**MLSPN.WC.0056**	surface open ocean (This study)	Marine
**MLSPN.WC.0074**	surface open ocean (This study)	Marine
**MLSPN.WC.0088**	surface open ocean (This study)	Marine
**MLSPN.WC.0098**	surface open ocean (This study)	Marine
**MLSPN.WC.0108**	surface open ocean (This study)	Marine
**MLSPN.WC.0128**	surface open ocean (This study)	Marine

Accession number	Source	Category
**MLSPN.WC.0148**	surface open ocean (This study)	Marine
**MLSPN.WC.0176**	surface open ocean (This study)	Marine
**MLSPN.WC.0188**	surface open ocean (This study)	Marine
**MLSPN.WC.0206**	surface open ocean (This study)	Marine
**MLSPN.WC.0254**	surface open ocean (This study)	Marine
**MLSPN.WC.0284**	surface open ocean (This study)	Marine
**MLSPN.WC.0304**	surface open ocean (This study)	Marine
**MLSPN.WC.0306**	surface open ocean (This study)	Marine
**MLSPN.WC.0326**	surface open ocean (This study)	Marine
**MLSPN.WC.0345**	surface open ocean (This study)	Marine
**MLSPN.WC.0365**	surface open ocean (This study)	Marine
**MLSPN.WC.0385**	surface open ocean (This study)	Marine
**MLSPN.WC.0406**	surface open ocean (This study)	Marine
**MLSPN.WC.0426**	surface open ocean (This study)	Marine
**MLSPN.WC.0446**	surface open ocean (This study)	Marine
**MLSPN.WC.0466**	surface open ocean (This study)	Marine
**MLSPN.WC.0486**	surface open ocean (This study)	Marine
**MLSPN.WC.506**	surface open ocean (This study)	Marine
**MLSPN.WC.526**	surface open ocean (This study)	Marine
**MLSPN.WC.546**	surface open ocean (This study)	Marine
**MLSPN.WC.566**	surface open ocean (This study)	Marine
**MLSPN.WC.2494**	surface open ocean (This study)	Marine
**MLSPN.WC.2614**	surface open ocean (This study)	Marine
**MLSPN.WC.2683**	surface open ocean (This study)	Marine
**MLSPN.WC.2891**	surface open ocean (This study)	Marine
**MLSPN.WC.2999**	surface open ocean (This study)	Marine
**ERR440973**	Coastal	Marine
**ERR440972**	Coastal	Marine
**ERR440930**	Coastal	Marine
**ERR440922**	Coastal	Marine
**ERR440904**	Coastal	Marine
**ERR440899**	Coastal	Marine
**ERR440863**	Coastal	Marine
**ERR440808**	Coastal	Marine
**ERR440804**	Coastal	Marine
**ERR440974**	Coastal	Marine
**ERR440932**	Coastal	Marine
**ERR440830**	Coastal	Marine

Supplementary Methods

Calculation of the significant wave height.

Modelled 6-hourly fields of both significant wave height (SWH) and its wind-wave component (SWHw) with a spatial resolution of 0.75x0.75 degree were used to explore the relationships with the microbial abundances (Supplementary Fig. 2). SWH fields were directly obtained from the ERA-INTERIM Reanalysis [1] while SWHw fields were estimated using the parameterization of the wind speed fields from ERA-Interim at the same resolution [2]. The SWH and SWHw values at the closest grid points to the 118 sampling points are listed in Supplementary Table 1.

Determination of total chlorophyll-*a*.

Chlorophyll-*a* concentration was determined fluorometrically, following Yentsch & Menzel [3] acetone extraction of chlorophyll-*a* retained in GF/F filters after filtering 200-500 ml samples [4]. The values of chlorophyll-*a* are shown in Supplementary Table 1.

Calculation of wet deposition.

The abundance of bioaerosols was not measured in the rainwater collected during the Malaspina Expedition. Therefore, the wet deposition fluxes of bioaerosols were estimated by multiplying the prokaryotic and eukaryotic abundances by the aerosol phase washout ratio (W_A),

$$F_{Wet,p} = W_A C_P \qquad \text{(S1)}$$

$$F_{Wet,e} = W_A C_E \qquad \text{(S2)}$$

where C_P and C_E are the concentrations of prokaryotes and eukaryotes, respectively.

The values of W_A were estimated for eight rain events from which the measurements of the concentrations of polycyclic aromatic hydrocarbons (PAHs) were available, and used in ten rain events previous to bioaerosol measurements. Atmospheric PAHs partitioning between the gas and aerosol phase. In the aerosol phase, PAHs are sorbed exclusively to carbonaceous particles (organic and soot carbon) [5]. Therefore, in this work, PAHs are used as proxy of the dynamics of organic matter aerosols in the atmosphere. PAHs were measured in rain ($C_{RAIN,PAH}$), the aerosol phase ($C_{A,PAH}$), and

the gas phase ($C_{G,PAH}$) during the Malaspina expedition as reported elsewhere [6]. The concentration of PAHs in rain water can be modelled by Jurado et al. [7],

$$C_{RAIN,PAH} = C_{A,PAH}W_A + C_{G,PAH}W_G \qquad \text{(S3)}$$

where W_G is the washout ratio of gas phase PAHs, which equals the inverse of the dimension-less Henry's law constant (1/H') [7]. Therefore, the rain event specific W_A values can be obtained from equation [S3].

The median of W_A for the ten rain events was of 4.5 10^5, and ranged from 3.9 10^3 to 2 10^7. These W_A values are of similar magnitude to those described previously in the literature for organic compounds [7], and are taken in this work as proxy of the efficiency of precipitation for scavenging bioaerosols. The values of $F_{Wet,P}$ and $F_{Wet,E}$ are shown in Supplementary Table 1.

Supplementary References

1. Dee, D. P. *et al.* The ERA-Interim reanalysis: configuration and performance of the data assimilation system. *Q. J. R. Meteorol. Soc.* 137, 553–597 (2011).
2. Pierson Jr, W. J. Comment on 'Effects of sea maturity on satellite altimeter measurements' by Roman E. Glazman and Stuart H. Pilorz. *J. Geophys. Res.* 96, 4973–4977 (1991).
3. Yentsch, C. S. & Menzel, D. W. A method for the determination of phytoplankton chlorophyll and phaeophytin by fluorescence. in *Deep Sea Research and Oceanographic Abstracts* 10, 221–231 (Elsevier, 1963).
4. Estrada, M. in *Expedición de circunnavegación Malaspina 2010. Cambio global y exploración de la biodiversidad del océano. Libro blanco de métodos y técnicas de trabajo oceanográfico* 399–406 (CSIC, 2012).
5. Lohmann, R. & Lammel, G. Adsorptive and Absorptive Contributions to the Gas-Particle Partitioning of Polycyclic Aromatic Hydrocarbons: State of Knowledge

and Recommended Parametrization for Modeling. *Environ. Sci. Technol.* 38, 3793–3803 (2004).

6. González-Gaya, B. *et al.* High atmosphere–ocean exchange of semivolatile aromatic hydrocarbons. *Nat. Geosci.* doi:10.1038/NGEO2714
7. Jurado, E. *et al.* Wet Deposition of Persistent Organic Pollutants to the Global Oceans. *Environ. Sci. Technol.* 39, 2426–2435 (2005).
